# A Lrp/AsnC Family Transcriptional Regulator Lrp Is Essential for the Pathogenicity of *Dickeya oryzae*

**DOI:** 10.1111/mpp.70100

**Published:** 2025-06-07

**Authors:** Xiaoyan Wu, Qunyi Chen, Huidi Liu, Weihan Gu, Yizhen Deng, Lian‐Hui Zhang, Zhibin Liang

**Affiliations:** ^1^ Guangdong Province Key Laboratory of Microbial Signals and Disease Control, Integrative Microbiology Research Centre South China Agricultural University Guangzhou China

**Keywords:** cell wall‐degrading enzymes, *Dickeya oryzae*, Lrp/AsnC family, pathogenicity, zeamines

## Abstract

*Dickeya oryzae* causes severe soft rot diseases in a range of important crops. To understand its complicated pathogenic mechanisms, we tried to identify the key virulence regulators through transposon mutagenesis. This led to the identification of a member of the Lrp/AsnC family transcriptional regulators in *D. oryzae* EC1, designated as Lrp. Phenotype analyses showed that Lrp positively regulated biofilm formation and the production of zeamines, proteases and polygalacturonases, but negatively regulated bacterial swimming motility. Deletion of *lrp* caused a drastic attenuation in bacterial virulence, indicating that Lrp is a key regulator in the modulation of *D. oryzae* pathogenicity. We further showed that the transcription of the *lrp* gene was negatively regulated by the transcriptional regulators SlyA, Fis and OhrR, and the transcriptional expression of *tzpA*, *ohrR* and *fis* was positively modulated by Lrp. Moreover, we demonstrated that Lrp can directly bind to the promoter regions of *zmsA*, *zmsK*, *prtG*, *prtX*, *pehK*, *pehX*, *fis*, *tzpA* and *ohrR*. DNase I footprinting assay determined that Lrp was capable of binding to a specific site (5′‐GTGTAATTATGGGCGTGCTCCGGG‐3′) in the promoter of *zmsA*. Furthermore, we found that four amino acid residues of Lrp, L20, L23, G111 and T146, are essential to the biological function of Lrp. Overall, this study demonstrated that Lrp is an essential virulence modulator in *D. oryzae* and suggested that Lrp can be a potent target for controlling the soft rot diseases caused by *D. oryzae* .

## Graphical abstract

Lrp is crucial for regulating the pathogenicity of *Dickeya oryzae* by forming a hierarchical regulatory network with other transcriptional regulatory factors and directly modulating the expression of virulence genes.

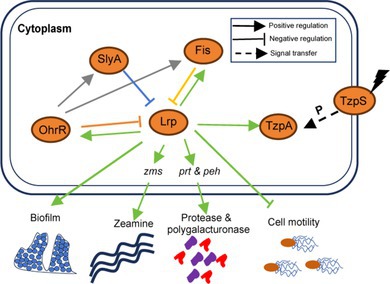

## 1 Introduction

*Dickeya oryzae* is a plant pathogen that causes diseases in important monocotyledonous and dicotyledonous crops (Li et al. 2020; Brady et al. 2012), including rice (Bez et al. 2021), banana (Hu et al. 2018) and potato (Chen et al. 2019). This pathogen poses a great threat to agricultural production and causes huge economic losses. The broad host range of *D. oryzae* is attributed to its ability to produce a set of virulence factors such as phytotoxic zeamines (Liao et al. 2014), motility (Shi et al. 2019; Chen et al. 2020) and cell wall‐degrading enzymes (CWDEs, including polygalacturonases, pectin lyases, proteases and cellulases) (Chen, Li, et al. 2022; Lv et al. 2019).

Zeamine is a family of polyketide molecules including zeamine, zeamine I and zeamine II (Masschelein, Clauwers, Stalmans, et al. 2015). As potent phytotoxins, zeamines are key virulence determinants of *D. oryzae* (Zhou et al. 2011; Cheng et al. 2013). In addition, zeamines are highly effective antibiotics with broad‐spectrum activity against a variety of microorganisms (Masschelein, Clauwers, Awodi, et al. 2015; Liao et al. 2015). The *zmsABCDEGIJKN* genes in *D. oryzae* are responsible for production of zeamines (Zhou et al. 2015). Inactivation of *zmsA* abolishes zeamine production and drastically attenuates the virulence of *D. oryzae* (Zhou et al. 2011). Therefore, zeamine production represents an effective biomarker for identification of virulence regulators in *D. oryzae* .

Expression of virulence genes in *D. oryzae* is regulated by at least three quorum‐sensing (QS) systems, which are mediated by *N*‐acyl homoserine lactone (AHL) (Hussain et al. 2008), putrescine (PUT) (Shi et al. 2019) and the virulence factor modulating (VFM) molecule (Nasser et al. 2013; Lv et al. 2019). In addition, several transcriptional regulatory proteins, including the MarR family regulator SlyA and OhrR (Zhou et al. 2016; Lv, Chen, et al. 2022), and the Fis family regulator Fis (Lv et al. 2018), are known to be involved in the regulation of *D. oryzae* virulence. SlyA and OhrR are positive regulators modulating the production of zeamines and CDWEs, biofilm formation and bacterial virulence, but they play negative roles in the regulation of bacterial swimming motility (Zhou et al. 2016; Lv, Chen, et al. 2022). In contrast, Fis positively regulates the production of zeamines and CDWEs, cell motility and biofilm formation, but negatively modulates the production of extracellular polysaccharides (EPS) (Lv et al. 2018). Among them, OhrR seems to play a central role by positively regulating the transcriptional expression of *slyA* and *fis* (Lv, Chen, et al. 2022). Besides, the GacA‐GacS type two‐component system TzpS‐TzpA also plays a key role in the positive regulation of zeamines biosynthesis and the virulence of *D. oryzae* (Chen, Li, et al. 2022). These findings indicate that *D. oryzae* has evolved complicated regulatory mechanisms in modulating its physiology and pathogenesis.

To further decipher the sophisticated regulatory mechanisms that govern the virulence of *D. oryzae*, we conducted a large‐scale transposon mutagenesis screening by using zeamine production as a biomarker. Among the zeamine‐defective mutants, we found that transposon insertion of a previously uncharacterised gene encoding an Lrp/AsnC family transcription factor in *D. oryzae* EC1 led to complete loss of zeamine production under the experimental conditions used in this study. This gene shares about 79% and 100% similarity to the leucine‐responsive regulatory protein (Lrp) regulator of *Escherichia coli* at the nucleic acid and amino acid levels, respectively, and hence was designated as *lrp*. We found that Lrp regulated a variety of biological functions including production of zeamines and CDWEs, cell motility and biofilm formation, all of which contributed to the pathogenicity of *D. oryzae* EC1. We further demonstrated that Lrp regulates these virulence traits by directly regulating not only the transcriptional expression of *ohrR*, *fis* and *tzpA*, which are known to encode key regulators of *D. oryzae* virulence, but also the transcriptional expression of virulence genes.

## 2 Results

### 2.1 Lrp Is Involved in Regulation of Zeamine Production in *D. oryzae* EC1

**FIGURE 1:**
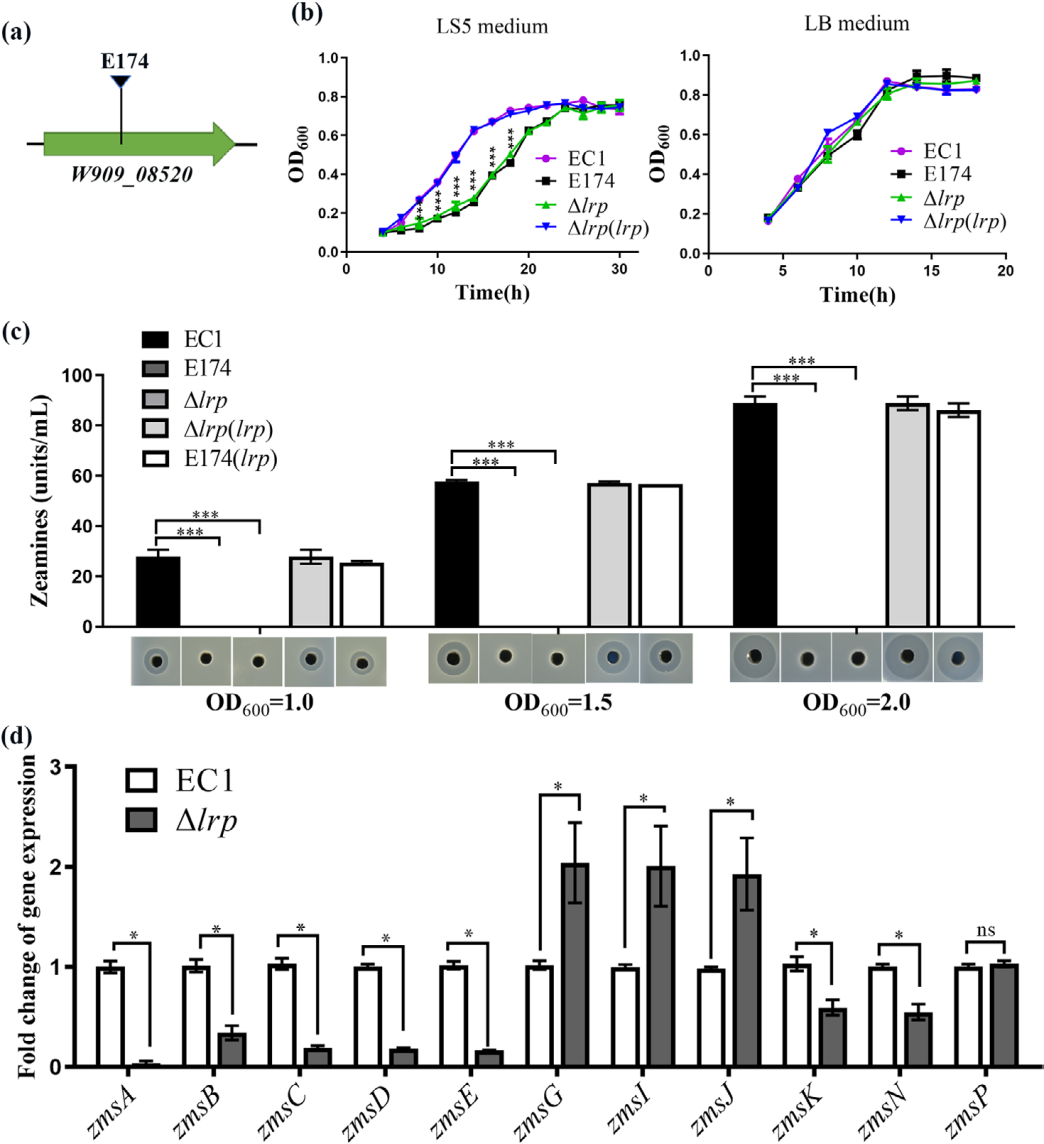
Mutation of *lrp* in *Dickeya oryzae* results in slow growth and defect in zeamine production. (a) The site of transposon insertion in mutant E174. The arrow indicates the position of transposon insertion in *lrp*. (b) Growth of strain EC1 and its derivatives in LS5 and Luria Bertani (LB) medium. (c) Zeamine production of strain EC1 and its derivatives in LS5 medium. (d) The transcriptional levels of *zms* cluster genes in strain EC1 and Δ*lrp*. The 16S rRNA gene served as a reference gene to normalise the gene expression. Fold change was calculated by using the 2^−ΔΔ*C*t^ method. The experiments were repeated at least three times. Data are presented as mean ± standard error (*n* = 3). Statistical analyses were performed using either one‐way ANOVA (b) (c) or the permutation test (d) versus strain EC1. *p < 0.05, ***p < 0.001, ns, not significant (*p* > 0.05).

To identify the regulators associated with *D. oryzae* virulence, we conducted a large‐scale screening of the Tn*5* transposon insertion library of strain EC1 (Zhou et al. 2016; Liang, Huang, et al. 2023), and looked for mutants with decreased zeamine production based on a previously established zeamine production assay method (Zhou et al. 2016). Among about 40,000 transposon insertional mutants screened in our study, we found 204 transposon insertion mutants that showed altered zeamine production. The FPNI‐PCR (Fusion Primer and Nested Integrated PCR) and DNA sequencing analysis indicated that most of them have a Tn*5* insertion in the same gene. Among them, we found the transposon insertion mutants 19, 35 and E72 have a Tn*5* insertion in the previously identified genes contributing to the positive regulation of zeamine production, namely *tzpA* (19), *vfmI* (35) and *zmsA* (E72) (Chen, Li, et al. 2022; Lv et al. 2019; Zhou et al. 2011) (Figure S1). In addition, we also found E174, a transposon mutant with a Tn*5* insertion in an uncharacterised gene in *D. oryzae* EC1, displayed a complete loss of zeamine production under a culturing condition (LS5 medium) optimised for zeamine production (Liao et al. 2014). The FPNI‐PCR and DNA sequencing results indicated the Tn*5* insertion in the mutant E174 was located in a gene encoding an Lrp/AsnC family transcription factor (NCBI accession no. *W909_08520*) (Figure 1a). We performed a BlastP search in NCBI by using the amino acid sequence of this gene as a subject. The result indicated that this gene is *lrp*, as it shares about 79% and 100% sequence similarity at the nucleic acid and amino acid levels, respectively, with the canonical *lrp* of *E. coli* MG1655 (NCBI accession no. *b0889*) (Kroner et al. 2019). This gene was named *lrp* hereafter.

To understand the relationship between Lrp and the growth of *D. oryzae* EC1, transposon mutant E174, in‐frame deletion mutant Δ*lrp*, and the complemented strains Δ*lrp*(*lrp*) and E174(*lrp*) were generated and their growth curves were determined. The results showed that the growth patterns of wild‐type EC1, transposon mutant E174, in‐frame deletion mutant Δ*lrp*, and the complemented strains Δ*lrp*(*lrp*) and E174(*lrp*) were similar in Luria Bertani (LB) medium. In contrast, in LS5 medium, E174 and Δ*lrp* grew much slower than strain EC1, but they caught up at the late stationary phase (Figure 1b). We then determined the zeamine production of strain EC1, E174, Δ*lrp*, Δ*lrp*(*lrp*) and E174(*lrp*) cultured in LS5 medium, with samples collected at an optical density at 600 nm (OD_600_) about 1.0, 1.5 and 2.0, respectively. We found that E174 and Δ*lrp* did not produce a detectable level of zeamines at any bacterial growth stage, while Δ*lrp*(*lrp*) and E174(*lrp*) produced a similar level of zeamines comparable to that of strain EC1 (Figure 1c). These results indicated that Lrp confers bacterial growth and zeamine production in *D. oryzae* EC1.

In the *zms* cluster, *zmsABCDE* are required for the biosynthesis of zeamine II (Zhou et al. 2011; Masschelein, Clauwers, Stalmans, et al. 2015), while *zmsGIJKN* contribute to the biosynthesis of two zeamine II derivatives, zeamine and zeamine I. *zmsLMPQR* may encode transporter proteins for zeamine production (Cheng et al. 2013; Masschelein et al. 2013; Masschelein, Clauwers, Stalmans, et al. 2015; Zhou et al. 2015). To further elucidate how Lrp regulates zeamine production, we determined the transcriptional levels of *zms* cluster genes, including *zmsABCDEGIJKN*, in strain EC1 and Δ*lrp* by reverse transcription‐quantitative PCR (RT‐qPCR) under LS5 culture conditions. The results showed that, unlike the putative zeamine transporter‐encoding gene *zmsP* showing comparable expression levels in both strain EC1 and Δ*lrp*, the transcriptional levels of *zmsA*, *zmsB*, *zmsC*, *zmsD*, *zmsE*, *zmsK* and *zmsN* were significantly decreased in Δ*lrp* compared to EC1, whereas the transcriptional levels of *zmsG*, *zmsI* and *zmsJ* were dramatically increased (Figure 1d). These findings indicate that Lrp regulates zeamine production by modulating the expression of key genes required for zeamine biosynthesis.

### 2.2 Lrp Controls the Polygalacturonase and Protease Production in *D. oryzae* EC1

**FIGURE 2:**
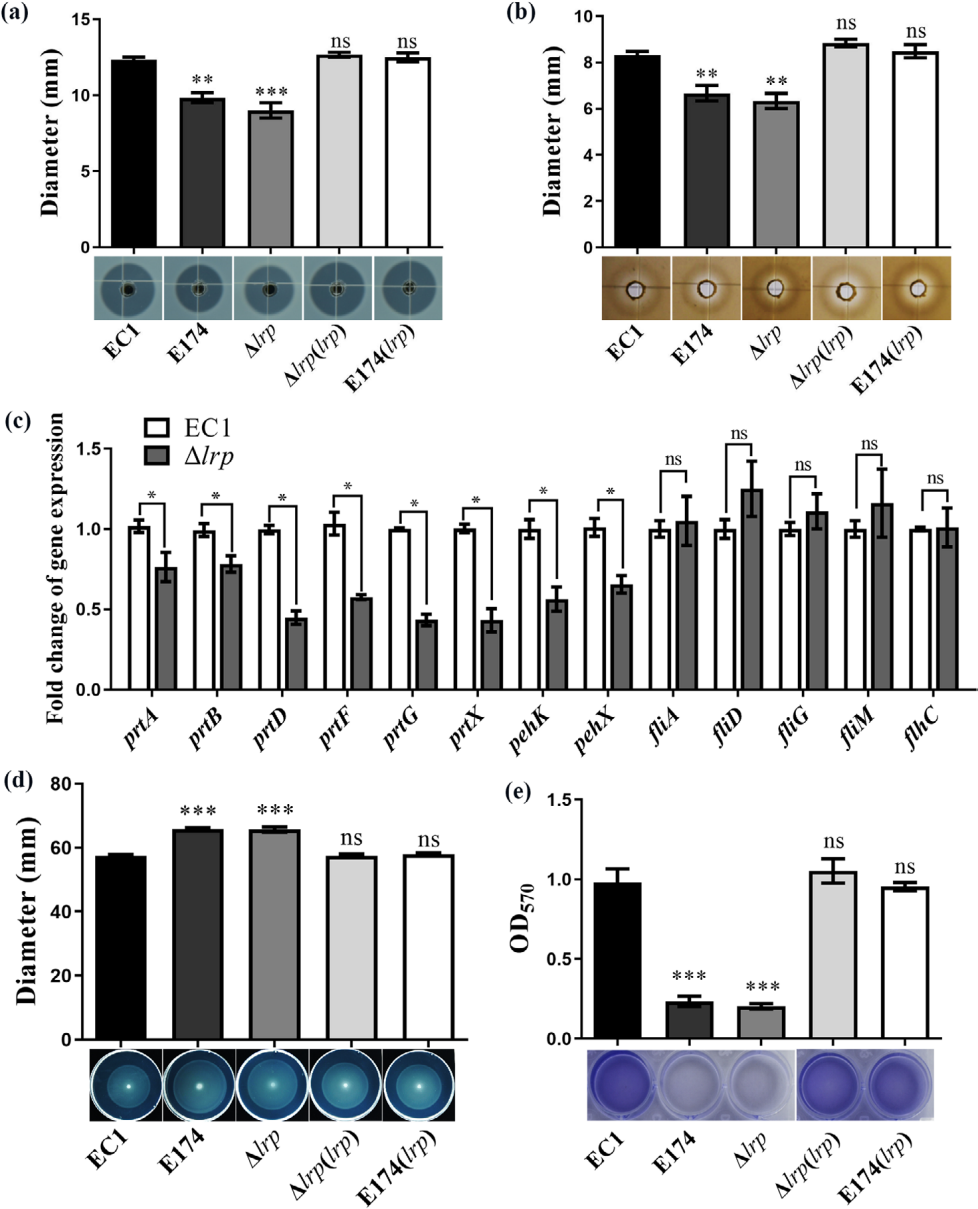
Mutation of *lrp* reduces polygalacturonase production, protease production and biofilm formation, and enhances swimming motility. Polygalacturonase production (a), protease production (b), swimming motility (d) and biofilm formation (e) of *Dickeya oryzae* EC1 and its derivatives. (c) Reverse transcription‐quantitative PCR analysis of *peh*, *prt* and flagella biosynthesis genes in mutant Δ*lrp* compared to wild‐type EC1. The 16S rRNA gene served as a reference gene to normalise the gene expression. Fold change was calculated by using the 2^−ΔΔ*C*t^ method. The experiments were repeated at least three times. Data are presented as mean ± standard error (*n* = 3). Statistical analyses were performed using either one‐way ANOVA (a), (b), (d) and (e), or the permutation test (c) versus strain EC1. *p < 0.05, **p < 0.01, ***p < 0.001, ns, not significant (*p* > 0.05).

Previous research showed that the production of CWDEs is required for the full virulence of *D. oryzae* EC1 (Chen, Hu, et al. 2022; Lv et al. 2019). To gain insight into the regulatory spectrum of Lrp, we detected the production of cellulase, pectinase, protease and polygalacturonase in strain EC1 and its derivatives after culturing these strains in LB medium. The results showed that the production of protease and polygalacturonase was significantly decreased in the mutants E174 and Δ*lrp*, compared with Δ*lrp*(*lrp*), E174(*lrp*) and EC1 (Figure 2a,b). However, the deletion of *lrp* did not seem to affect the cellulase and pectinase production (Figure S2).

In *D. oryzae* EC1, *pehK* and *pehX* are genes predicted to encode polygalacturonase, while *prtGABX* and *prtDF* encode proteases and protease secretion‐associated proteins, respectively (Zhou et al. 2015). To determine whether the transcription of these genes is dependent on Lrp, RT‐qPCR analysis was performed. The results showed that the transcriptional levels of *pehK*, *pehX*, *prtA*, *prtB*, *prtD*, *prtF*, *prtG* and *prtX* were significantly reduced in the mutant Δ*lrp* compared to those in strain EC1 (Figure 2c), which unveiled the positive regulatory role of Lrp on the transcriptional expression of genes associated with the production of protease and polygalacturonase.

### 2.3 Lrp Regulates the Swimming Motility and Biofilm Formation in *D. oryzae* EC1

We then investigated the effect of Lrp on the swimming motility of *D. oryzae* EC1, which is another key virulence determinant responsible for invasion and systemic infection (Chen et al. 2019; Lv, Ye, et al. 2022). The results indicated that E174 and Δ*lrp* displayed enhanced bacterial motility compared to wild‐type EC1, while Δ*lrp*(*lrp*) and E174(*lrp*) had a comparable swimming motility to EC1 (Figure 2d), suggesting that Lrp negatively regulates swimming motility. RT‐qPCR results showed that deletion of *lrp* did not alter the expression level of *fliA*, *flhC*, *fliD*, *fliG* and *fliM* (Figure 2c), which are the key genes involved in flagella biosynthesis (Lv, Chen, et al. 2022; Lv et al. 2018), indicating that Lrp regulation on swimming motility is not dependent on transcriptional regulation of flagella biosynthesis genes.

Biofilm is associated with the virulence of *D. oryzae* due to its roles in attachment on host surface and resistance to biotic stresses (Lv, Ye, et al. 2022). We measured the biofilm formation of strain EC1 and its derivatives and found that loss of a functional Lrp (E174 and Δ*lrp*) compromised biofilm formation, which could be restored by in‐trans expression of *lrp* (Figure 2e). The results suggested that Lrp plays a critical role in the regulation of biofilm formation.

### 2.4 Identification of the Key Residues of Lrp Essential for Its Biological Function

**FIGURE 3:**
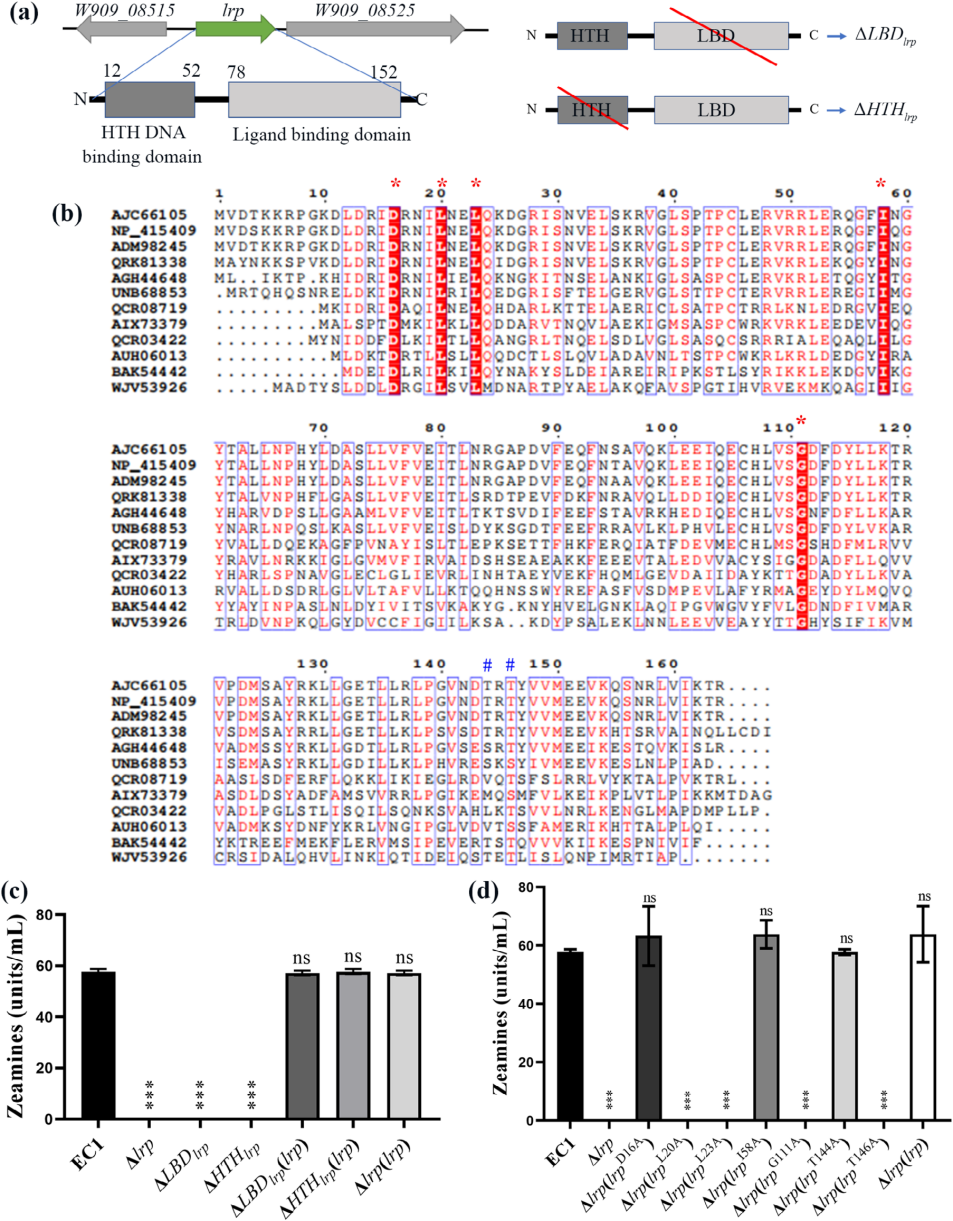
Characterisation of the functional domains and conserved amino acid residues of Lrp. (a) Schematic diagrams showing the genetic organisation of *lrp* with its upstream and downstream genes, the domain structures of Lrp, and the domains of Lrp in Δ*LBD*_*lrp*_ and Δ*HTH*_*lrp*_. Domain structures were predicted using the SMART program. (b) Sequence alignment analysis of Lrp in strain *Dickeya oryzae* EC1 (NCBI accession no. AJC66105) and its homologues in *Escherichia coli* MG1655 (NCBI accession no. NP_415409), *Dickeya dadantii* 3937 (NCBI accession no. ADM98245), *Shewanella* sp. LZH‐2 (NCBI accession no. QRK81338), *Paraglaciecola psychrophila* 170 (NCBI accession no. AGH44648), *Pseudomonas syringae* pv. *tagetis* ICMP 4091 (NCBI accession no. UNB68853), *Brenneria rubrifaciens* 6D370 (NCBI accession no. QCR08719), *Pantoea* sp. PSNIH2 (NCBI accession no. AIX73379), *Brenneria nigrifluens* ATCC 13028 (NCBI accession no. QCR03422), *Prodigiosinella confusarubida* ATCC 39006 (NCBI accession no. AUH06013) *Sulfolobus tokodaii* 7 (NCBI accession no. BAK54442), *Prodigiosinella aquatilis* LS101 (NCBI accession no. WJV53926). *Highly conserved sites; ^#^The functional sites detected in Lrp of *S. tokodaii* 7. (c) Zeamine production by wild‐type EC1 and its derivatives in LS5 medium at OD_600_ = 1.5. (d) Zeamine production of wild‐type EC1 and its derivatives in LS5 medium. The experiments were repeated at least three times. Data are presented as mean ± standard deviation (*n* = 3). Statistical analyses were performed using one‐way ANOVA versus strain EC1. ***p < 0.001, ns, not significant (*p* > 0.05).

According to the Simple Modular Architecture Research Tool (SMART), Lrp consists of an HTH DNA‐binding domain (HTH, 12th to 52th amino acid residues) and a ligand‐binding domain (LBD, 78th to 152th amino acids residues) (Figure 3a). To determine the potential roles of HTH and LBD domains in the biological functions of Lrp, we generated the domain‐deletion mutants, Δ*LBD*_*lrp*_ and Δ*HTH*_*lrp*_ (Figure 3a), and determined their role in zeamine production. The results showed that neither Δ*LBD*_*lrp*_ nor Δ*HTH*_*lrp*_ could produce a detectable level of zeamines (Figure 3c). These results suggested that both LBD and HTH domains are indispensable for the regulatory role of Lrp in *D. oryzae* EC1.

To determine the key amino acid residues of Lrp essential for its regulatory function, the amino acid sequence of Lrp in *D. oryzae* EC1 (NCBI accession no. AJC66105) was compared with its relatives in *E. coli* MG1655 (NCBI accession no. NP_415409), *Dickeya dadantii* 3937 (NCBI accession no. ADM98245), *Shewanella* sp. LZH‐2 (NCBI accession no. QRK81338), *Paraglaciecola psychrophila* 170 (NCBI accession no. AGH44648), *Pseudomonas syringae* pv. *tagetis* ICMP 4091 (NCBI accession no. UNB68853), *Brenneria rubrifaciens* 6D370 (NCBI accession no. QCR08719), *Pantoea* sp. PSNIH2 (NCBI accession no. AIX73379), *Brenneria nigrifluens* ATCC 13028 (NCBI accession no. QCR03422), *Prodigiosinella confusarubida* ATCC 39006 (NCBI accession no. AUH06013), *Sulfolobus tokodaii* 7 (NCBI accession no. BAK54442) and *Prodigiosinella aquatilis* LS101 (NCBI accession no. WJV53926). We found that Lrp in *D. oryzae* EC1 has a high level of amino acid sequence identity and similarity with the Lrp proteins in *E. coli* MG1655 and *D. dadantii* 3937. They form a monophyletic clade in the phylogenetic tree, whereas the Lrp homologues with a relatively lower level of amino acid sequence identity and similarity from other genera were more distantly related (Figure S3). The alignment analysis unveiled five highly conserved amino acid residues, D16, L20, L23, I58 and G111 (Figure 3b). In addition, two residues T144 and T146 were also included in the subsequent analysis because they correspond to the T132 and T134 residues of the Lrp in *S. tokodaii* 7 (Grp) (Figure 3b), which have been reported to be involved in reinforcing the quaternary structure and regulatory function (Kumarevel et al. 2008).

To test whether these seven amino acid residues are required for the Lrp function in *D. oryzae* EC1, we substituted these seven residues with alanine (A) to generate complemented strains with mutation, that is Δ*lrp*(*lrp* ^D16A^), Δ*lrp*(*lrp* ^L20A^), Δ*lrp*(*lrp* ^L23A^), Δ*lrp*(*lrp* ^I58A^), Δ*lrp*(*lrp* ^G111A^), Δ*lrp*(*lrp* ^T144A^) and Δ*lrp*(*lrp* ^T146A^). Subsequently, we examined zeamine production of EC1 and the above seven strains. The results showed that in‐trans expression of *lrp* ^L20A^, *lrp* ^L23A^, *lrp* ^G111A^ or *lrp* ^T146A^ in Δ*lrp* did not restore zeamine production (Figure 3d). However, zeamine production of Δ*lrp* could be restored by in‐trans expression of *lrp* ^D16A^, *lrp* ^I58A^ or *lrp* ^T144A^ (Figure 3d). These results indicated that L20, L23, G111 and T146 are critical for the regulatory function of Lrp in *D. oryzae* EC1.

### 2.5 Lrp Is Essential for the Pathogenicity of *D. oryzae* EC1

**FIGURE 4:**
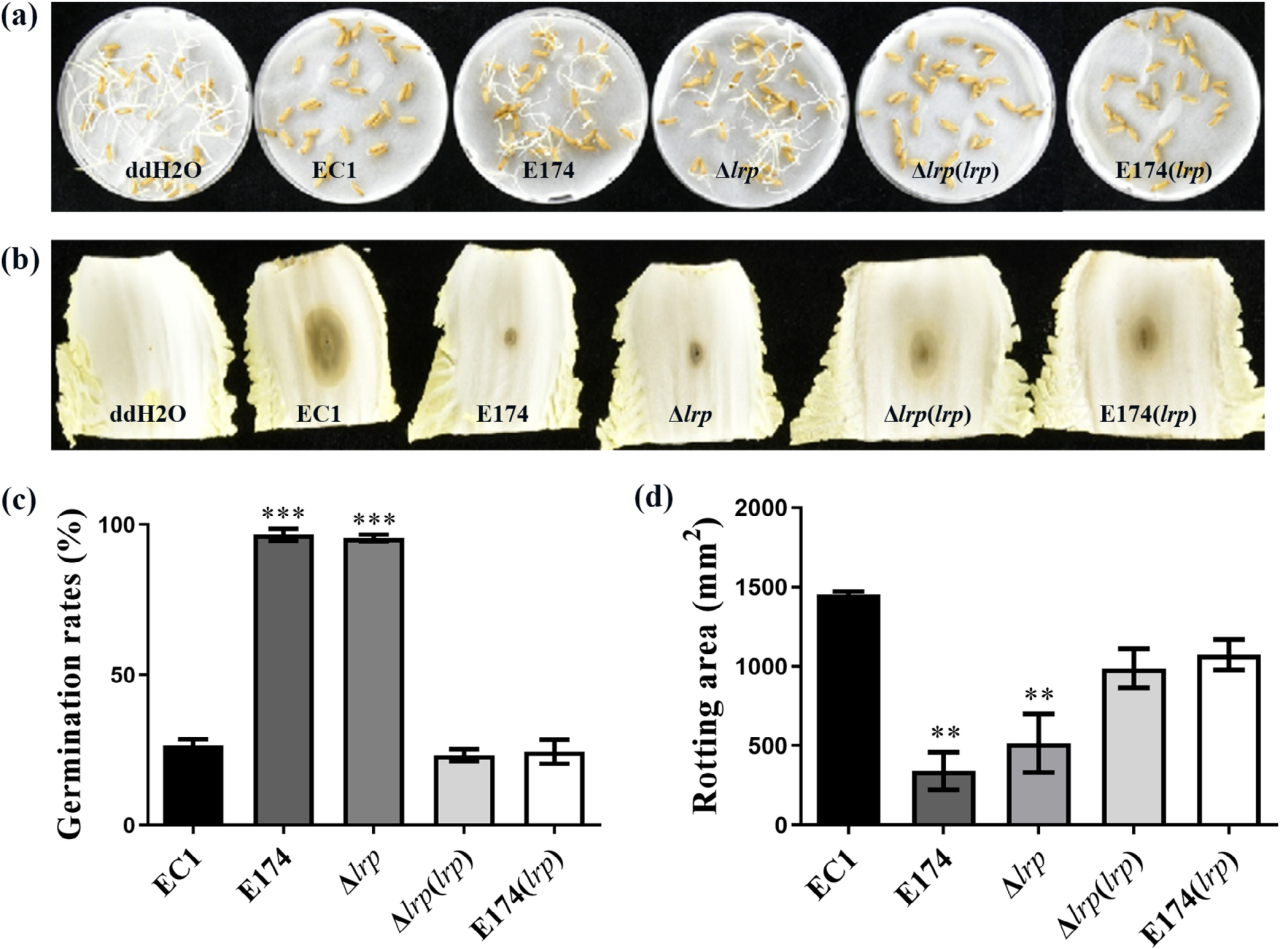
Inactivation of *lrp* reduces the pathogenicity of *Dickeya oryzae* EC1 on rice seed, Chinese cabbage and radish. Rice seed germination (a) and germination rate (c) of rice seeds treated with strain EC1 and its derivatives. Soft rot symptoms on Chinese cabbage (b) and rotting area on radish roots (d) treated with strain EC1 and its derivatives. The experiments were repeated at least three times. Data are presented as mean ± standard error (*n* = 30) (c) or (*n* = 3) (d). Statistical analyses were performed using one‐way ANOVA versus strain EC1. **p < 0.01, ***p < 0.001.

To investigate the role of Lrp on the virulence of *D. oryzae* EC1, we inoculated rice seeds with the cell cultures of strain EC1 and its derivatives and assessed the rice seed germination rate at 7 days post‐inoculation (dpi). The results showed that mutation of *lrp* drastically attenuated bacterial virulence. The germination rate of rice seeds treated with Δ*lrp* or E174 was about 97% (Figure 4a,c), which was much higher than the germination rate of rice seeds treated with strain EC1, Δ*lrp*(*lrp*) or E174(*lrp*) (Figure 4a,c). Similarly, Chinese cabbage leaves and radish tuber slices inoculated with the Δ*lrp* or E174 had substantially reduced rotting areas compared to those inoculated with strain EC1, Δ*lrp*(*lrp*) or E174(*lrp*) (Figure 4b,d). Overall, these results demonstrated that Lrp plays a significant role in the modulation of *D. oryzae* virulence on host plants.

### 2.6 Lrp Forms a Regulatory Network With Other Regulatory Factors

**FIGURE 5:**
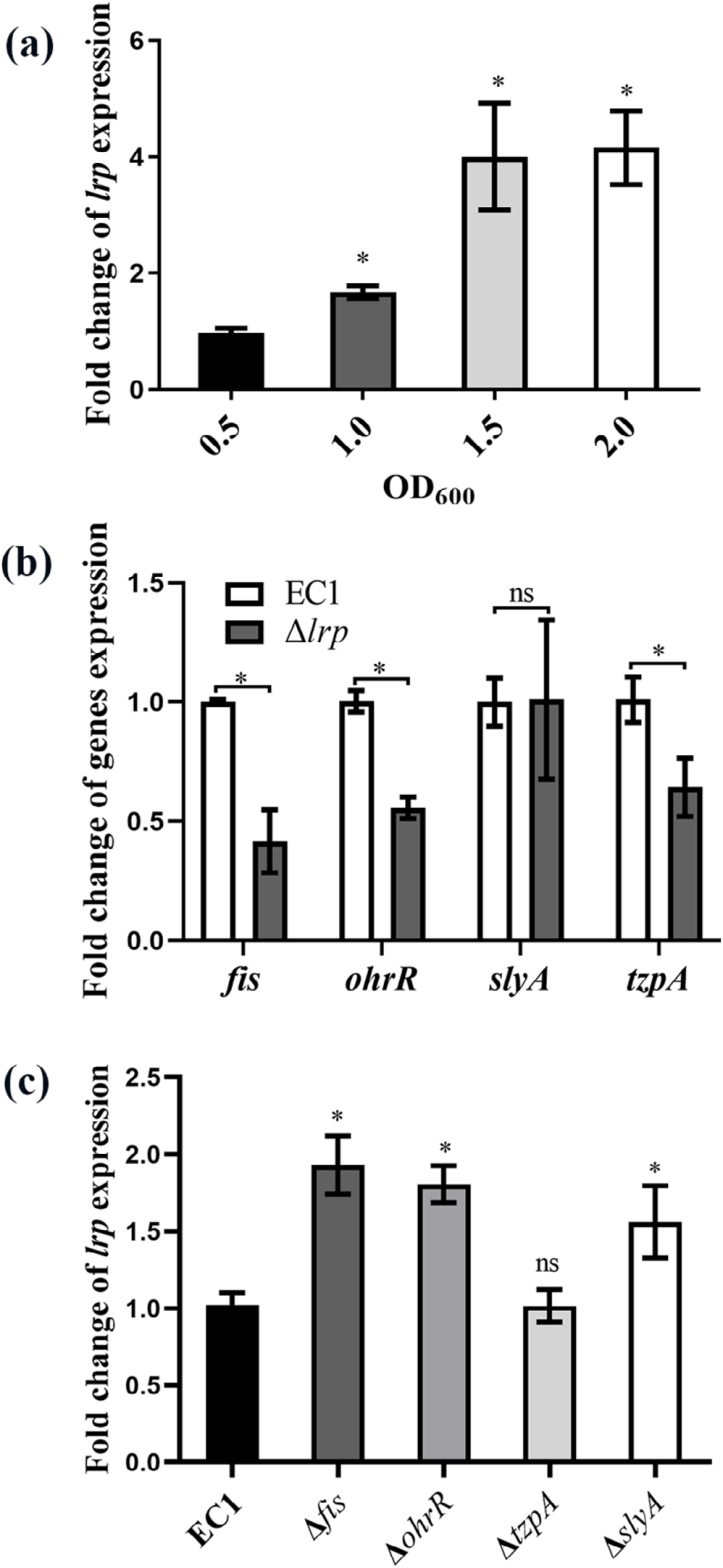
The interaction between Lrp and the regulators OhrR, SlyA, TzpA and Fis. (a) Reverse transcription‐quantitative PCR (RT‐qPCR) analysis of the expression of *lrp* in the wild‐type EC1 cultured in Luria Bertani medium at different bacterial growth stages. (b) RT‐qPCR analysis of *fis*, *ohrR*, *slyA* and *tzpA* transcription in mutant Δ*lrp* compared to wild‐type EC1. (c) RT‐qPCR analysis of *lrp* in Δ*fis*, Δ*ohrR*, Δ*slyA* and Δ*tzpA* compared to wild‐type EC1. The experiments were repeated at least three times. The 16S rRNA gene was used as a reference gene to normalise the gene expression. Fold change was calculated by using the 2^−ΔΔ*C*t^ method. Data are presented as mean ± standard deviation (*n* = 3). Statistical analyses were performed using the permutation test versus OD_600_ = 0.5 (a) or versus strain EC1 (b, c). *p < 0.05, ns, not significant (*p* > 0.05).

Given the important role of Lrp in regulation of bacterial physiology and virulence, it is interesting to find how the *lrp* expression is modulated. We firstly tested whether the expression of *lrp* depends on bacterial population density. The RT‐qPCR analysis unveiled that the transcriptional level of *lrp* in strain EC1 increased along with bacterial growth and became steady at the time when the OD_600_ of bacterial cell culture reached 1.5 (Figure 5a).

Previously, studies unveiled that several transcription regulators, including SlyA, Fis, OhrR and TzpA, positively regulate zeamine biosynthesis (Zhou et al. 2016; Lv et al. 2018; Lv, Chen, et al. 2022; Chen, Li, et al. 2022). Given the fact that Lrp can regulate zeamine production, we speculated that Lrp might be part of the regulatory network consisting of these regulators. To validate this speculation, we measured the transcriptional level of *slyA*, *fis*, *ohrR* and *tzpA* in Δ*lrp* in comparison to that in strain EC1. The results showed that, with the exception of *slyA*, the transcriptional levels of the other three regulatory genes, *ohrR*, *fis* and *tzpA*, were reduced in Δ*lrp* compared to those in strain EC1 (Figure 5b). On the other hand, the expression of *lrp* was also found to be influenced by some of the above regulators. The transcriptional level of *lrp* was significantly increased in Δ*fis*, Δ*ohrR*, Δ*slyA*, but not in Δ*tzpA*, compared to that in strain EC1 (Figure 5c). The results indicate that the transcriptional expression of *lrp* is negatively regulated by Fis, SlyA and OhrR, while Lrp plays a positive regulatory role in controlling the expression of *ohrR*, *fis* and *tzpA*.

We further investigated whether there was a regulatory relationship between QS systems and Lrp by analysing the transcriptional level of the QS genes *vfmE*, *expI* and *speA*, which encode the production of corresponding QS signals VFM, AHL and PUT, respectively (Lv et al. 2019; Hussain et al. 2008; Shi et al. 2019), and the transcriptional level of *lrp* in the QS mutants Δ*vfmE*, Δ*expI* and Δ*speA*. The results showed that *lrp* transcription did not seem to be significantly regulated by the VFM, AHL or PUT QS systems, and the expression of these QS genes was not affected by Lrp either (Table [Link], [Link]).

### 2.7 Lrp Directly Binds to the Promoters of Virulence Genes

**FIGURE 6:**
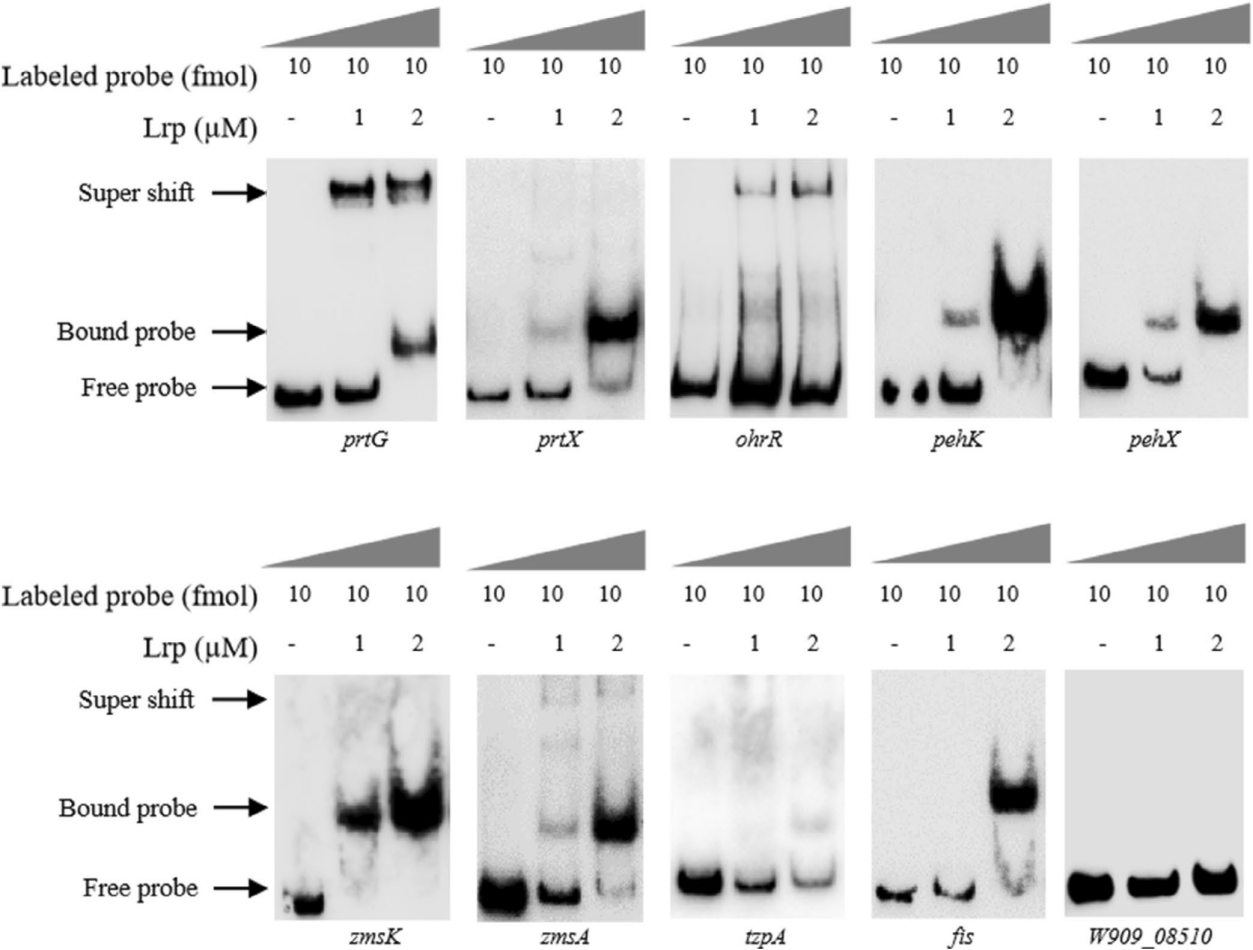
Lrp directly interacts with the promoters of *zmsA*, *zmsK*, *prtG*, *prtX*, *pehK*, *pehX*, *fis*, *ohrR* and *tzpA*. Electrophoretic mobility shift assay was performed by incubation of the labelled promoter DNA probes (10 fmol) with 1 or 2 μM Lrp. Free probe and bound probe were indicated by arrows. The probe constructed from the promoter region of *W909_08510* served as a negative control. The experiments were repeated at least three times.

Next, we investigated whether Lrp regulates the transcription of target genes by directly binding to their promoters. Lrp protein was expressed by using a prokaryotic expression system (Figure S4) and purified for electrophoretic mobility shift assay (EMSA). The results showed that Lrp caused an obvious band shift of the biotin‐labelled promoter fragments of *zmsA*, *zmsK*, *prtG*, *prtX*, *pehK*, *pehX*, *fis*, *tzpA* and *ohrR* (Figure 6), indicating that Lrp can directly and indirectly control the transcriptional expression of the genes for production of zeamines, protease and polygalacturonase in *D. oryzae* EC1.

**FIGURE 7:**
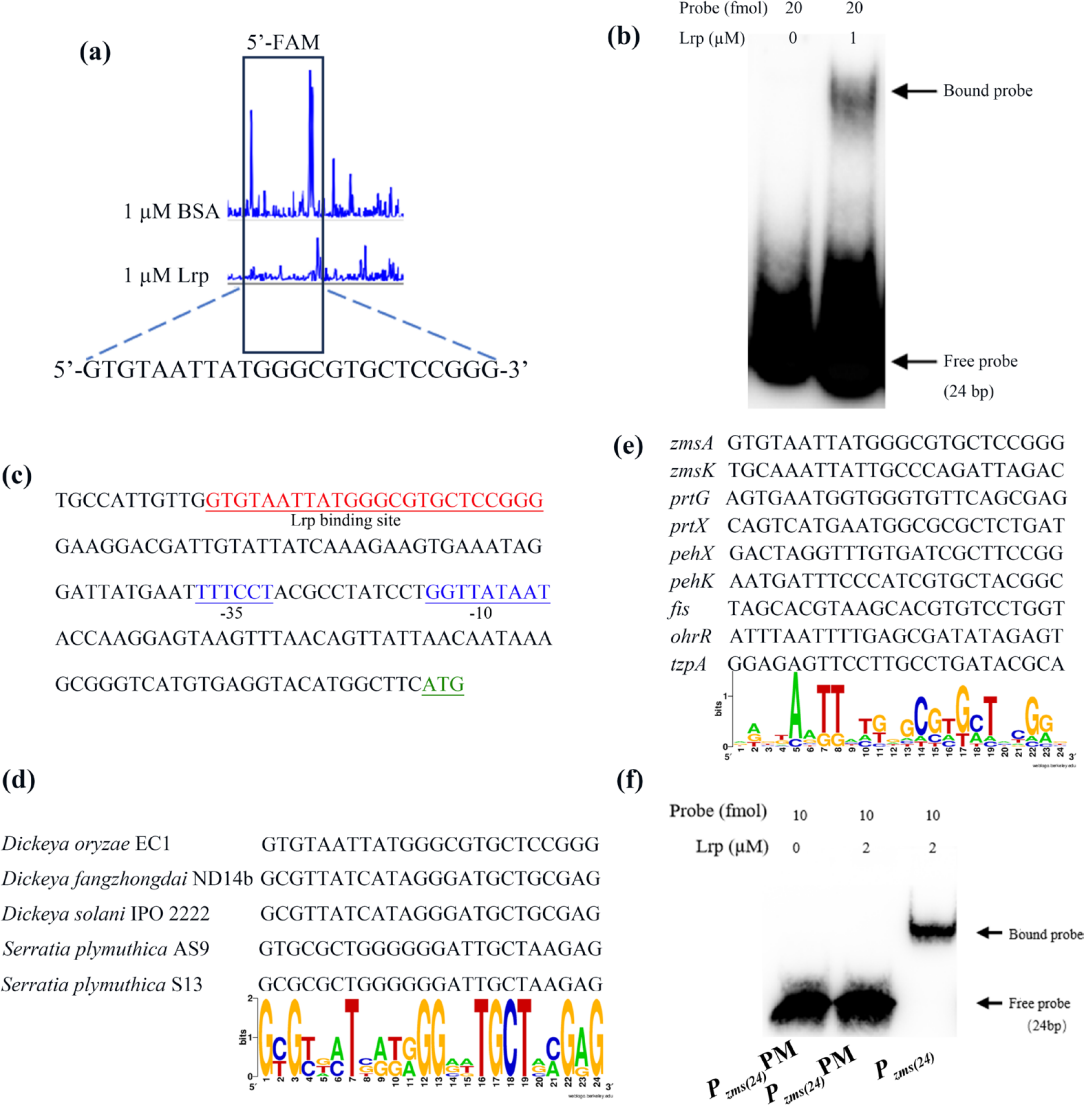
Identification of the Lrp‐binding region in *zmsA* promoter. (a) DNase I footprinting assay was performed using Lrp and the promoter region of *zmsA* labelled with 5′‐FAM. (b) Electrophoretic mobility shift assay of Lrp binding to a 24‐bp DNA fragment *P*_*zmsA*(24)_ containing putative Lrp‐binding site identified in the DNase I footprinting assay (a). Labelled DNA probes (20 fmol) were incubated with 1 μM Lrp. The free and bound probes were indicated by arrows. (c) Lrp‐binding site in the *zmsA* promoter region. The Lrp‐binding site (red font) and start codon (ATG) of *zmsA* gene (green font) were underlined. The experiments were repeated at least three times. (d) Identification of the conserved sites for Lrp binding in the promoters of *zmsA* homologues from *Dickeya fangzhongdai* ND14b, *Dickeya solani* IPO 2222, *Serratia plymuthica* S13, *S. plymuthica* AS9 and *Dickeya oryzae* EC1 by SeqLogo analysis. (e) Identification of the conserved sites for Lrp binding by aligning the *P*_*zmsA*(24)_ with the sequences of *PzmsK*, *PprtG*, *PprtX*, *PpehK*, *PpehX*, *PohrR*, *PtzpA* and *Pfis*. (f) Mutation of the putative conserved Lrp‐binding sites in *P*_*zmsA*(24)_ abolished Lrp binding. *P*_*zmsA*(24)_ (5′‐GTGTAATTATGGGCGTGCTCCGGG‐3′) was mutated to construct the *P*_*zmsA*(24)_PM (5′‐GTGTGAAAAGAGTGAGAGACCACG‐3′) to determine the specific Lrp‐binding sites in *P*_*zmsA*(24)_.

To define the specific binding motif of Lrp, DNase I footprinting assay was carried out by using the *zmsA* promoter (*P*_*zmsA*_) labelled with FAM (5′) as described previously (Liang, Lin, et al. 2023). The results unveiled a 24 bp motif (5′‐GTGTAATTATGGGCGTGCTCCGGG‐3′) in *P*_*zmsA*_, designated as *P*_*zmsA*(24)_, that was specifically bound by Lrp (Figure 7a,c). For validation, the *P*_*zmsA*(24)_ motif was synthesised and labelled with biotin for EMSA by using purified Lrp protein. The results showed that Lrp caused a clear band shift of the biotin‐*P*_*zmsA*(24)_ (Figure 7b), confirming that this 24‐bp fragment is the specific binding motif for Lrp (Figure 7c).

To determine whether the *P*_*zmsA*(24)_ motif was conserved in other species or other gene promoters in strain EC1, we analysed their DNA sequences on the website (https://weblogo.berkeley.edu/logo.cgi). By alignment of the above *P*_*zmsA*(24)_ motif with the promoters of the *zmsA* homologues in different bacterial species, including *D. fangzhongdai* ND14b, *D. solani* IPO 2222, *Serratia plymuthica* AS9 and *S. plymuthica* S13, we found 11 highly conserved nucleotides in the sequence 5′‐GyGydmTbrkrGGvdTGCTvmGrG‐3′ (Figure 7d). Given that Lrp homologues are also highly conserved (over 98% similarity) in these bacterial species containing the *zms* cluster, these findings suggest that the Lrp‐dependent regulation of zeamine production might be well conserved in these zeamine‐producing bacterial species. Besides, alignment of *P*_*zmsA*(24)_ motif with the 200‐bp promoter sequences including *PzmsK*, *PprtG*, *PprtX*, *PpehK*, *PpehX*, *PohrR*, *PtzpA* and *Pfis* directly bound by Lrp unveiled a sequence with conserved nucleotides: 5′‐ddnkAdTTwTGrGCGwGCThyGGn‐3′ (Figure 7e). The conserved nucleotides were then mutated in the sequence of *P*_*zmsA*(24)_ to generate a mutated probe *P*_*zmsA*(24)_PM (5′‐GTGTGAAAAGAGTGAGAGACCACG‐3′). The EMSA experiment showed that Lrp did not interact with *P*_*zmsA*(24)_PM (Figure 7f), indicating the binding of Lrp to *P*_*zmsA*(24)_ relied on the putative conserved Lrp‐binding sites in *P*_*zmsA*_. These findings suggest that these conserved nucleotides might be of critical importance to the interaction between Lrp and the promoters of target genes in *D. oryzae* EC1.

## 3 Discussion

Lrp and its homologue AsnC represent a family of widely conserved transcriptional regulators that modulate distinct biological functions in prokaryotes (Deng et al. 2011; Knoten et al. 2011; Aguilar‐Barajas et al. 2013; Ho et al. 2017; Modrzejewska et al. 2021). In *E. coli* , Lrp serves as a global factor controlling about 200 genes, which regulate functions including responses to osmotic stress, nutrient limitation and high concentrations of organic acids (Tani et al. 2002), and influences pilus formation, amino acid synthesis, transport and degradation, and porin biosynthesis (Haney et al. 1992; Willins et al. 1991). There are seven putative Lrp/AsnC family members in *D. oryzae* ; however, their biological functions have not yet been investigated. In this study, we found that inactivation of the *lrp* gene in *D. oryzae* EC1 caused a complete loss of zeamine production (Figure 1c), decreased polygalacturonase and protease production (Figure 2a,b), enhanced cell motility (Figure 2d), reduced biofilm formation (Figure 2e) and drastically attenuated bacterial pathogenicity (Figure 4). These findings established that Lrp is a vital regulator modulating bacterial physiology and virulence.

**FIGURE 8:**
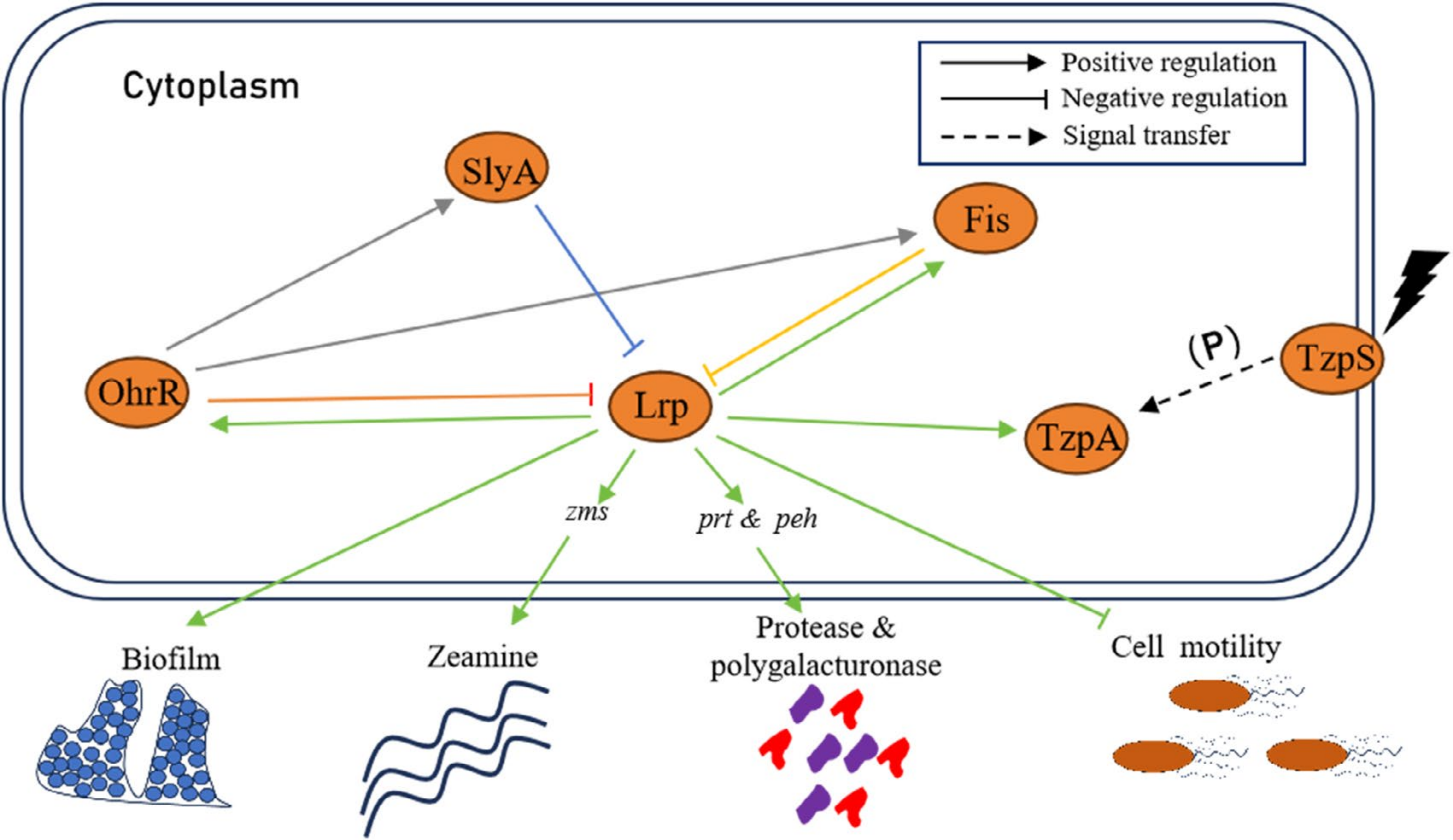
Schematic representation of the Lrp, TzpA/S, OhrR, Fis and SlyA regulatory network in *Dickeya oryzae* EC1. The *lrp* gene is negatively regulated by transcriptional regulators SlyA, Fis and OhrR, and transcriptional expression of *tzpA*, *ohrR* and *fis* is positively modulated by Lrp. Lrp can directly interact with the promoters of *zmsA*, *zmsK*, *prtG*, *prtX*, *pehK* and *pehX* to regulate the production of zeamines, protease and polygalacturonase.

This is the first report that a Lrp family protein is associated with the regulation of the zeamine production. Our previous study showed that inactivation of *zmsA* led to complete loss of zeamine production, abolished the antimicrobial activity and drastically decreased the bacterial virulence of *D. oryzae* EC1 (Zhou et al. 2011). Significantly, expression of *zmsA* decreased significantly in Δ*lrp* (Figure 1d), which explains the abolished zeamine production phenotype in Δ*lrp*. Our previous studies unveiled several regulatory proteins involved in the regulation of zeamine production, including SlyA (Zhou et al. 2011), OhrR (Lv, Chen, et al. 2022), Fis (Lv et al. 2018) and TzpA (Chen, Li, et al. 2022). In contrast to the complete loss of zeamine production in the *lrp* mutant, disruption of the genes resulted in only partial reduction in zeamine production. RT‐qPCR analysis validated the vital role of Lrp in controlling the transcriptional expression of zeamine biosynthesis genes (Figure 1d). Interestingly, this study unveiled that the transcriptional expression of *lrp* was negatively regulated by Fis, SlyA and OhrR (Figure 5c), and Lrp positively regulated the expression of *ohrR*, *fis* and *tzpA* (Figure 5b). These results suggest that zeamine biosynthesis in *D. oryzae* is controlled by multiple regulators, including TzpA, OhrR, SlyA, Fis and Lrp, which form a sophisticated regulatory network to coordinate the production of zeamines and other virulence factors. The regulatory interconnection of Lrp and the previously determined regulators TzpA, Fis, SlyA and OhrR is shown in Figure 8.

Previous study showed that Lrp homologues could interact with either glutamine or leucine as a signal ligand (Kumarevel et al. 2008). Crystal structure analysis of the Lrp homologue Grp from *S. tokodaii* 7, which is a glutamine receptor protein, led to the identification of three key amino acid residues, Y77, T132 and T134. Of these, Y77 is crucial for ligand binding and the residues T132 and T134 affect protein oligomerisation for DNA binding (Kumarevel et al. 2008). The amino acid sequence alignment indicates that the Lrp of *D. oryzae* EC1 is highly similar to the leucine receptor protein Lrp from *E. coli* but is dissimilar to the glutamine receptor Grp of *S. tokodaii* 7 (Figures 3b and S3). We conducted site‐directed mutagenesis to identify the key functional residues of Lrp. Domain structure analysis showed that the Lrp of *D. oryzae* EC1 contains two functional domains, HTH and LBD, both of which were required for the regulatory function of Lrp (Figure 3c). Sequence alignment of Lrp homologues identified five highly conserved amino acid residues (D16, L20, L23, I58, G111) and two conserved residues (T144 and T146) corresponding to the residues T132 and T134 of Grp (Figure 3b), respectively, that confer Grp function (Kumarevel et al. 2008). Among them, the residues D16, L20, L23 and I58 are located in the HTH domain, while residues G111, T144 and T146 are located in the LBD domain. It was noteworthy that L20, L23, G111 and T146 were indispensable for zeamine production (Figure 3d), suggesting that they are the key amino acid residues for Lrp function. The residue T134 of the glutamine receptor Grp in *S. tokodaii* 7 was shown to play a role in stabilising the dimer–dimer interface and strengthening the quaternary protein structure (Kumarevel et al. 2008). This highlights that its counterpart in Lrp of *D. oryzae* EC1, that is T146, may also contribute to maintaining the protein structure of Lrp of *D. oryzae* EC1.

In this study, we found that Lrp recognised a 24‐bp DNA sequence (5′‐GTGTAATTATGGGCGTGCTCCGGG‐3′) in the promoter of *zmsA* (Figure 7). However, the Lrp promoter binding motif identified in this study appears different from the previously reported Lrp‐binding motifs in other bacterial species, including Lrp of *Vibrio vulnificus* (with 95% similarity at amino acid level) binding consensus sequence mkCrTTkwAyTsTG (Ho et al. 2017), the predicted Lrp of *Pseudomonas aeruginosa* PAO1 (with 40% similarity at amino acid level) binding sequence 5′‐CCTGATAAAAAAGG‐3′ (Modrzejewska et al. 2021), and the deduced Lrp of *Pyrococcus horikoshii* (with 50% similarity at amino acid level) binding sequence 5′‐ATAAAATTTTTAT‐3′ (Koike et al. 2004). Given the Lrp homologues may differ substantially in amino acid similarity (Unoarumhi et al. 2016), variations in their binding sequences are expected.

The relationship between environmental signals and Lrp has gained much attention in different bacterial species. The amino acid leucine in the environment is considered to be linked to the action and function of Lrp (Chen, Rosner, et al. 2001; Chen, Hao, et al. 2001). Lrp is thought to exist in multimer states, and leucine promotes Lrp to form octamers in order to modulate Lrp function (Chen and Calvo 2002). In *Shigella*, Lrp may bind to the leucine from the colon, and thus induce the expression of a small RNA SsrV for activation of virulence genes (Li et al. 2024). In addition to leucine, alanine, methionine, isoleucine, histidine and threonine also affect the function of Lrp proteins (Lange et al. 2012; Hart et al. 2011; Ihara et al. 2017). Bacteria may assess the nutritional status of the current environment through the response of Lrp to exogenous amino acids, thereby controlling gene expression (Cho et al. 2008). The plant host produces a large variety of amino acids to synthesise protective natural products against pathogen infection (Zeier 2013). Phytopathogens may enhance the expression of the virulence genes by sensing these amino acid signals through Lrp proteins. We speculate that Lrp protein may be a key mediator for *D. oryzae* EC1 to sense host‐derived amino acid signals and initiate its infection.

In summary, this study unveiled that the transcriptional regulator Lrp plays a crucial regulatory role in controlling zeamine biosynthesis, biofilm formation and bacterial virulence. Identification of the key amino acid residues for Lrp biological functions, as well as the conserved Lrp‐binding sequence, not only enriches our understanding of the regulatory spectra and species‐specific features of the widely conserved Lrp/AsnC family regulators but also presents a potent target for controlling the disease caused by *D. oryzae* .

## 4 Experimental Procedures

### 4.1 Bacterial Strains, Primers, Media and Growth Conditions

The bacterial strains and plasmids used in this study are listed in Table [Link], [Link], and primers are in Table [Link], [Link]. *E. coli* strains were routinely cultured at 37°C in LB medium described previously (Liang et al. 2019). *D. oryzae* EC1 and its derived strains were cultured at 28°C in LB medium, minimal medium (MM) and LS5 medium described previously (Liang et al. 2019). Antibiotics were added as necessary using the following concentrations: gentamycin (Gen) at 50 μg/mL; ampicillin (Amp) at 100 μg/mL; polymyxin B (pB) at 25 μg/mL; kanamycin (Kan) at 50 μg/mL; and streptomycin (Str) at 50 μg/mL.

### 4.2 Amino Acid Sequence Analysis

The similarity analysis of amino acid sequence was performed on the website (http://www.detaibio.com/sms2/ident_sim.html).

### 4.3 Construction of Transposon Mutagenesis

Construction of transposon mutagenesis was performed as described previously (Zhou et al. 2016; Liang, Huang, et al. 2023). Briefly, conjugal mating was performed by mixing the overnight culture of *D. oryzae* EC1 and *E. coli* S17‐1 containing pBT20 on LB agar plates and incubated at 28°C for 6 h, and transposon mutants were selected on the MM agar plates containing 50 μg/mL Gen and 50 μg/mL Kan.

### 4.4 Fusion Primer and Nested Integrated PCR Analysis

The DNA fragment of the flanking region of the Tn*5* transposon insertion site was amplified using the FPNI‐PCR method described previously, with nested primers listed in Table [Link], [Link] (Wang et al. 2011; Liang, Lin et al. 2023).

### 4.5 Construction of In‐Frame Deletion Mutants and Complementation

Construction of in‐frame deletion mutants was performed following the previously described method (Cheng et al. 2016). Briefly, the primers 1/2 and 3/4 listed in Table [Link], [Link] were used to amplify fragments flanking each gene. The two fragments were fused together using primers 1/4. The fusion fragments and the suicide plasmid pKNG101 were digested with the restriction endonuclease BamHI and then ligated together by T4 DNA ligase. The resulting constructs were transformed into *E. coli* CC118 (Chen, Hu, et al. 2022; Chen et al. 2020). To generate in‐frame deletion mutants, the tripartite mating method was carried out as described previously (Lv et al. 2018). Deletion mutants were screened on MM agar plates supplemented with 5% (vol/vol) sucrose and were confirmed by DNA sequencing.

To generate complemented strains, the primers listed in Table [Link], [Link] were used to amplify DNA fragments containing the sequence of the target genes. The DNA fragments were cloned to the plasmid pBBR1‐MCS4. The resultant constructs were transformed into *E*. *coli* DH5α and mobilised into mutants by conjugal tripartite mating, following the method described previously (Lv, Chen, et al. 2022). Point mutation in the complemented genes was performed by using the Mut Express II fast mutagenesis kit V2 (Vazyme Biotech). The desired construct was transformed into wild‐type EC1 or its derivatives by triparental mating following a previously described method (Chen, Li, et al. 2022).

### 4.6 Growth Curve Assay

To determine the growth curve of *D. oryzae* EC1 and its derivatives, an aliquot of 1 μL of overnight bacterial culture (adjusted to an OD_600_ = 1.2) was added to 150 μL LS5 medium or LB medium in each well of 96‐well cell culture plates. The cell culture plate was shaken at 200 rpm at 28°C, and the OD_600_ of the bacterial culture was measured every 2 h by the growth curve instrument Bioscreen C. The experiment was repeated at least three times.

### 4.7 Zeamine Production Bioassay

The zeamine bioassay plates were prepared by pouring 25 mL of LB agar into a 10 × 10 cm plate, and then overlaid with 7.5 mL of 1% agarose containing 10^8^ cells of *E. coli* DH5α. Wells of 5‐mm diameter were punched in the bioassay plates after solidification. An aliquot of 10 μL of overnight wild‐type EC1 and its derivatives culture (OD_600_ = 1.2) was added to 10‐mL LS5 medium in a 50‐mL tube and grown at 28°C (Liao et al. 2014) in order to collect the cell cultures with the same OD_600_. About 14, 17 and 22 h are required for the wild‐type strain EC1, complemented strains Δ*lrp*(*lrp*) and E174(*lrp*) to reach an OD_600_ of 1.0, 1.5 and 2.0, respectively, while about 15, 18 and 24 h are required for the transposon mutant E174 and in‐frame deletion mutant Δ*lrp* to reach an OD_600_ of 1.0, 1.5 and 2.0, respectively. The collected bacterial cultures were centrifuged at 13,000 *g* for 2 min, and an aliquot of 30 μL of supernatant was added to each well. The assay plates were incubated overnight at 37°C. The diameter of the visible clear zone surrounding the wells was measured. Data was analysed by the formula zeamines (units/mL) = 5 × [(*D* − 5) × 10]/3, where *D* is the width in mm of the inhibition zone surrounding the well. The experiment was repeated at least three times.

### 4.8 CWDE Activity Assay

The cellulase, pectate lyase, polygalacturonase and protease activities were measured using carboxymethyl cellulose sodium, polygalacturonic acid and skimmed milk as the substrates, respectively. CWDE activities of EC1 and its derivatives were measured following the method described previously (Chen et al. 2016). Briefly, approximately 25 mL of culture medium was poured into a 10 × 10 cm plate. Wells with a diameter of 5 mm were punched in the solidified medium in the plates. The wild‐type EC1 and its derivatives were cultured in LB medium to an OD_600_ of 1.2. An aliquot of 40 μL of bacterial culture was added to each well, and the plates were incubated overnight at 28°C. After incubation, polygalacturonase and pectate lyase assay plates were covered with 4 M HCl for 10 min. Cellulase assay plates were stained with 0.1% Congo red (wt/vol) and then decoloured with 1 M NaCl. The diameter of the visible clear zone surrounding the wells was measured to determine the activity of CWDEs. The experiments were repeated at least three times.

### 4.9 Biofilm Formation and Swimming Motility Assay

To determine swimming motility of *D. oryzae* EC1 and its derivatives, bacterial strains were grown in LB overnight. Subsequently, an aliquot of 1 μL of overnight bacterial culture (OD_600_ = 1.2) was added to the middle of 15 mL of Bacto tryptone agar plates (5 g NaCl, 10 g Bacto tryptone, 3 g agar per L). Plates were incubated overnight at 28°C. The diameter of the chemotactic zones was measured.

To determine biofilm formation of *D. oryzae* EC1 and its derivatives, overnight bacterial cultures (OD_600_ = 1.2) were diluted in YEB medium (5 g sucrose, 5 g yeast extract, 10 g tryptone, 5 g KCl, 0.5 g MgSO_4_·7H_2_O per L) at a ratio of 1:1000, and 1 mL of diluted cultures was added into each well of a 24‐well microtitre plate. The plate was shaken at 250 rpm at 28°C for 24 h. Bacterial cell culture was then removed, and an aliquot of 1.5 mL of 1% (wt/vol) crystal violet solution was added into each well and incubated at room temperature for 15 min. The dye was removed and the wells were washed with clean water three times and dried at room temperature. An aliquot of 1.5 mL of ethanol with acetic acid (4:1) was added to dissolve the remaining crystal violet. The amount of biofilm formation was established by detecting the absorbance of the dissolved solution at 570 nm.

### 4.10 Pathogenicity Assays for Chinese Cabbage and Radish

The radishes were washed and cut into 1 cm thick slices, while Chinese cabbage leaves were cut into 5 × 5 cm chips. An aliquot of 1 μL of overnight bacterial culture (adjusted to an OD_600_ = 1.2) was added into the middle of each radish slice and cabbage leaf. After incubating at 28°C for 16 h, the area of decay was measured (Lv, Chen, et al. 2022).

### 4.11 Pathogenicity Assay for Rice Seed Germination

*Dickeya oryzae* EC1 or its derivatives were cultured to OD_600_ = 1.0 and diluted 10^6^‐fold in double‐distilled water. A total number of 30 rice seeds were immersed in 5‐mL diluted bacterial solution and incubated at room temperature for 5 h. As a negative control, rice seeds were incubated in 5‐mL double‐distilled water. The rice seeds were washed with double‐distilled water three times before being transferred to a Petri dish with a moist 10‐cm filter paper and incubated at 28°C under 16/8 h light/dark conditions. The seed germination rate was calculated after incubation for 1 week.

### 4.12 RNA Purification

The overnight cell culture of *D. oryzae* EC1 or its derivatives was diluted to the fresh LB medium or LS5 medium at a ratio of 1:1000. Bacterial cells (OD_600_ = 0.8) were collected by centrifugation at 13,000 *g* at 4°C for 1 min, and RNA molecules were purified with the SV total RNA isolation system kit (Promega). The quantity of RNA was determined by using a NanoDrop ND100 spectrophotometer, and the integrity of the RNA was assessed by using an agarose gel. The FastKing RT kit (with gDNase) (Tiangen Biotech) was used for DNA elimination and cDNA synthesis (Liang, Lin, et al. 2023). DNA contamination was checked before the cDNA synthesis step of the FastKing RT kit by PCR, using the primer pairs targeting the *desB* gene in RT‐qPCR analysis (Liang et al. 2019).

### 4.13 RT‐qPCR Analysis

RT‐qPCR analysis was performed on the CFX 96 real‐time PCR equipment to analyse the expression of target genes with ChamQ Universal SYBR qPCR Master Mix (Vazyme Biotech). The primers listed in Table [Link], [Link] were designed using Primer Quest software. The amplification procedure was performed as follows: 95°C for 30 s; 95°C for 10 s, 60°C for 30 s, 40 cycles; 95°C for 15 s, 60°C for 60s, 95°C for 15 s. An aliquot of 1 μL of 10‐fold diluted cDNA sample synthesised from 1 μg RNA was added to each reaction system. Data were analysed using the 2^−ΔΔ*C*t^ method, as previously described (Livak and Schmittgen 2001). The 16S rRNA gene was served as a reference gene to normalise gene expression (Li et al. 2022; Liang, Huang, et al. 2023; Zhao et al. 2021).

### 4.14 Protein Expression and Purification of Lrp

The primer pair pGEX‐*lrp*‐F/R listed in Table [Link], [Link] was used to amplify DNA fragments of the coding sequence of the *lrp* gene. These resultant DNA fragments were cloned into plasmid pGEX‐6P‐1 to generate pGEX‐6p‐*lrp*. *E. coli* BL21 containing pGEX‐6p‐*lrp* was grown in 1 L of LB medium at 16°C overnight, with IPTG added to a final concentration of 0.5 mM for protein expression. The cells were collected by centrifugation at 2500 *g* for 20 min and then resuspended in 20‐mL phosphate‐buffered saline containing 200 μL of protease inhibitor cocktail (Sangon Biotech). The cell resuspension was sonicated on ice with SONICS GM 3200. The supernatant was collected by centrifugation at 13,000 *g* for 20 min at 4°C and filtered. The Lrp‐GST was purified following the protocol described previously (Lv et al. 2018). Prescission Protease (Smart Lifesciences) was used to remove the GST tag.

### 4.15 Protein–DNA Interaction Assay

The 200‐bp promoter DNA fragments of target genes were predicted on the website (http://www.softberry.com/berry.phtml) and acquired by PCR using the primers listed in Table [Link], [Link]. The EMSA Probe Biotin Labeling Kit (Beyotime) was used to label the promoter fragments. Approximately 10 fmol labelled fragment and 1 or 2 μM of Lrp protein were mixed and incubated at room temperature for 20 min. The protein–DNA complex and unbound free DNA fragments were separated by using TBE electrophoresis buffer on a 6% nondenaturing polyacrylamide gel and were developed with the chemiluminescent EMSA kit (Beyotime).

### 4.16 DNase I Footprinting Assay

The DNase I footprinting assay was carried out following the method described previously with minor modifications (Liang, Lin, et al. 2023). To generate DNA probes, the promoter region of *zmsA* was amplified with the primer pair *zmsA*‐F (5′‐FAM)/*zmsA*‐R (Table [Link], [Link]). Approximately 20 fmol DNA probe was incubated with 1 μM Lrp protein for 30 min at room temperature. After incubation, an aliquot of 10 μL of DNase I (Promega) was added and incubated for 1 min. The reaction was stopped by DNase Stop Solution in the RQ1 RNase‐Free DNase kit (Promega). Before ethanol precipitation, total DNA was purified by adding phenol/chloroform and dissolved in 10 μL double‐distilled water. Purified DNA samples were run on a 3730xl system, and Peak Scanner 2 (Applied Biosystems) was used to analyse data.

### 4.17 Statistical Analysis

Every experiment was performed at least three times, each sample with three replicates. To analyse significantly different values between wild‐type EC1 and its derivatives, one‐way ANOVA analysis of variance was performed by using GraphPad Prism software (ns, not significant *p* > 0.05, *p < 0.05, **p < 0.01, ***p < 0.001). As the data of fold change of gene expression do not satisfy Student's *t* test assumptions, a permutation test was performed with the SPSS statistics software (ns, not significant *p* > 0.05, *p < 0.05).

## Conflicts of Interest

The authors declare no conflicts of interest.

## Supporting information


Figure S1.

Figure S2.

Figure S3.

Figure S4.

Table S1.

Table S2.

Table S3.

## Data Availability

All data are contained within the manuscript, figures and Supporting Information.
