# Supplementary material for: A Lrp/AsnC Family Transcriptional Regulator Lrp Is Essential for the Pathogenicity of Dickeya oryzae
Source: Mol Plant Pathol. 2025 Jun 7;26(6):e70100. doi: 10.1111/mpp.70100 (PMC12145271; doi:10.1111/mpp.70100)
Supplement: Supplementary file 1 — Figure S1. [file MPP-26-e70100-s003.docx]

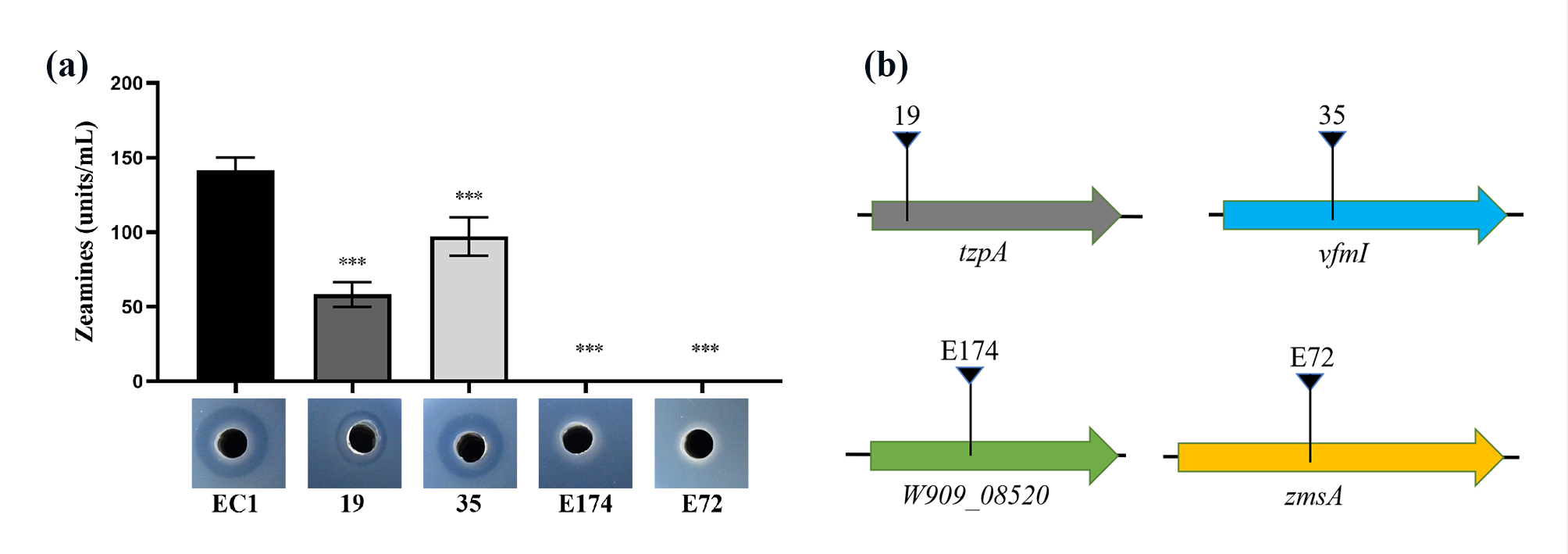


**Figure S1.** Identification of the transposon insertion mutant 19, 35, E174, and E72 with decreased zeamine production. (a) Zeamine production of wild-type strain EC1 and transposon insertion mutants cultured in LS5 medium when cell cultures reached an OD_600_ of 1.5. The experiments were repeated at least three times. Data are presented as mean  ±  standard error (*n* = 3). Statistical analyses were performed using one‐way ANOVA versus strain EC1. *** *p* < 0.001. (b) The site of transposon insertion in mutant 19, 35, E174, E72. The arrow indicates the position of transposon insertion in *tzpA* (19), *vfmI* (35), *W909_08520* (E174), and *zmsA* (E72).
