# Supplementary material for: A Lrp/AsnC Family Transcriptional Regulator Lrp Is Essential for the Pathogenicity of Dickeya oryzae
Source: Mol Plant Pathol. 2025 Jun 7;26(6):e70100. doi: 10.1111/mpp.70100 (PMC12145271; doi:10.1111/mpp.70100)
Supplement: Supplementary file 2 — Figure S2. [file MPP-26-e70100-s006.docx]

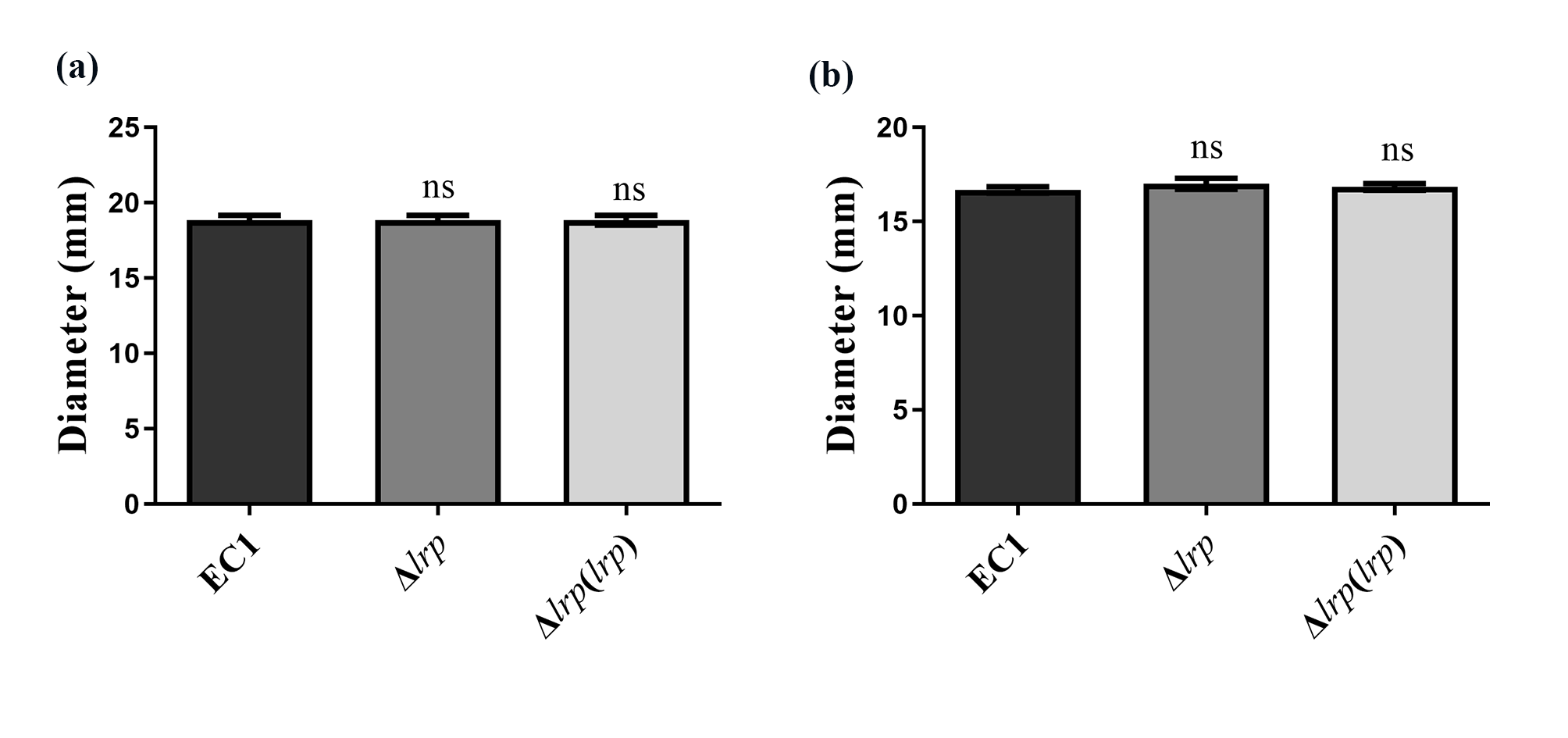


**Figure S2.** Lrp did not affect the production of pectinase and cellulase. Diameter of degradation zone for pectinase (a) and cellulase (b) in wild-type EC1 and its derivatives. The experiments were repeated at least three times. Data are presented as mean  ±  standard error (*n*  =  3). Statistical analyses were performed using one‐way ANOVA versus strain EC1. ns, not significant (*p* > 0.05).
