# Supplementary material for: A Lrp/AsnC Family Transcriptional Regulator Lrp Is Essential for the Pathogenicity of Dickeya oryzae
Source: Mol Plant Pathol. 2025 Jun 7;26(6):e70100. doi: 10.1111/mpp.70100 (PMC12145271; doi:10.1111/mpp.70100)
Supplement: Supplementary file 3 — Figure S3. [file MPP-26-e70100-s007.docx]

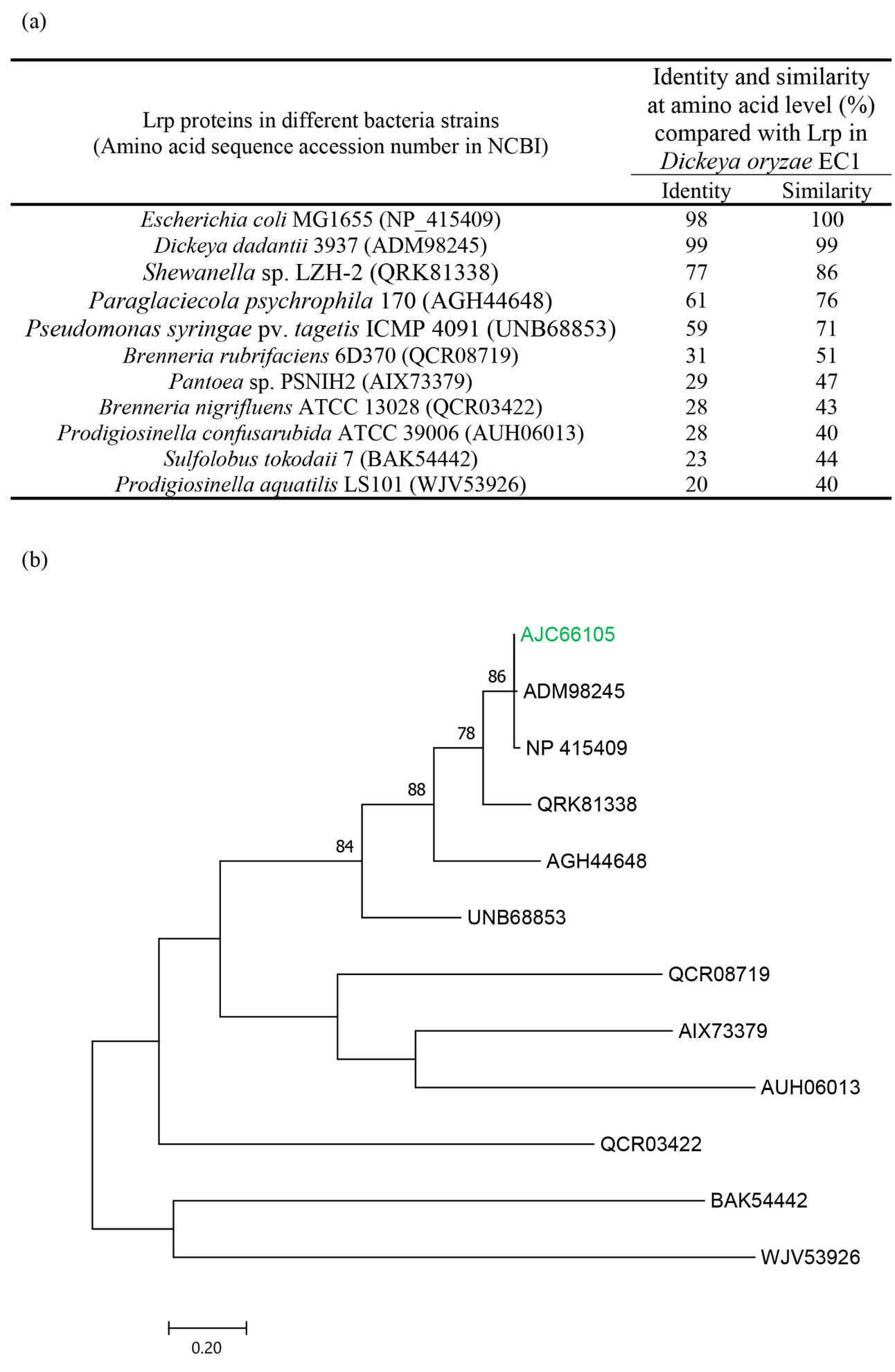


**Figure S3.** Comparison of Lrp proteins in different bacterial species. (a) Amino acid sequence identity or similarity compared with Lrp in *D. oryzae* EC1 for Lrp proteins in different bacterial species. (b) Phylogenic relationship of Lrp in *D. oryzae* EC1 and its homologs. The amino acid of Lrp in *D. oryzae* EC1 and its homologs were aligned by ClustalW and the phylogenic tress was constructed in MEGA7 (Kumar et al., 2016) with Maximum Likelihood method based on the best-fit model (“LG + G”) (Le and Gascuel, 2008). Bootstrap values higher than 70% are shown. Lrp in *D. oryzae* EC1 is marked with green.

**References**

Kumar, S., Stecher, G. & Tamura, K. (2016). MEGA7: Molecular Evolutionary Genetics Analysis version 7.0 for bigger datasets. *Molecular Biology and Evolution*, 33, 1870-1874.

Le, S.Q., & Gascuel, O. (2008). An improved general amino acid replacement matrix. *Molecular Biology and Evolution*, 25, 1307-1320.
