# Supplementary material for: A Lrp/AsnC Family Transcriptional Regulator Lrp Is Essential for the Pathogenicity of Dickeya oryzae
Source: Mol Plant Pathol. 2025 Jun 7;26(6):e70100. doi: 10.1111/mpp.70100 (PMC12145271; doi:10.1111/mpp.70100)
Supplement: Supplementary file 4 — Figure S4. [file MPP-26-e70100-s005.docx]

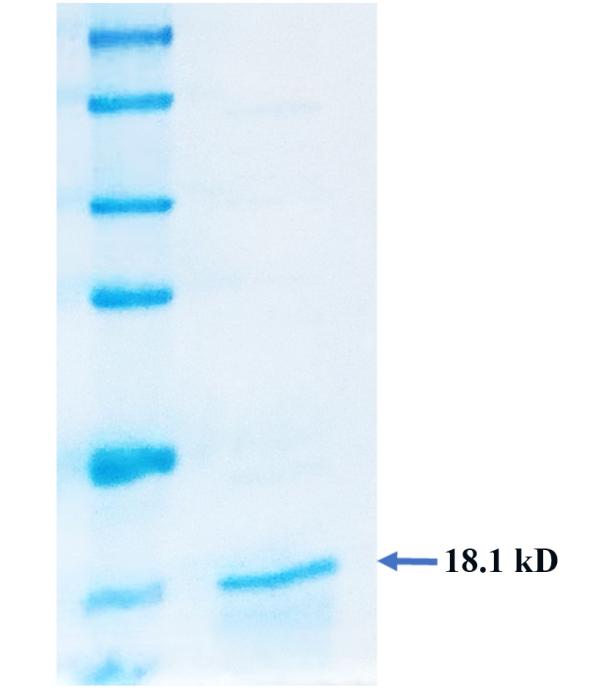


**Figure S4.** Polyacrylamide gel electrophoresis of the purified Lrp protein. The Lrp protein band was indicated by arrow.
