# Supplementary material for: A Lrp/AsnC Family Transcriptional Regulator Lrp Is Essential for the Pathogenicity of Dickeya oryzae
Source: Mol Plant Pathol. 2025 Jun 7;26(6):e70100. doi: 10.1111/mpp.70100 (PMC12145271; doi:10.1111/mpp.70100)
Supplement: Supplementary file 5 — Table S1. [file MPP-26-e70100-s001.docx]

**Table S1.** RT-qPCR analysis of target gene expression in the mutant Δ*lrp*, Δ*vfmE,* Δ*expI,* Δ*speA* compared to wild-type strain EC1

| Mutants | Target gene | Fold change (mean ± SD)^a^ |
| --- | --- | --- |
| Δ*lrp* | *vfmE* | 0.945 ± 0.001 |
|  | *expI* | 1.084 ± 0.001 |
|  | *speA* | 0.991 ± 0.001 |
| Δ*vfmE* | *lrp* | 1.006 ± 0.002 |
| Δ*expI* | *lrp* | 1.233 ± 0.002 |
| Δ*speA* | *lrp* | 1.211 ± 0.021 |

^a^The 16S rRNA gene was served as a reference gene to normalize the gene expression. Data is presented as mean ± standard deviation (*n* = 3), and fold change was calculated by using the 2^−ΔΔC^_T_ method.
