# Supplementary material for: A Lrp/AsnC Family Transcriptional Regulator Lrp Is Essential for the Pathogenicity of Dickeya oryzae
Source: Mol Plant Pathol. 2025 Jun 7;26(6):e70100. doi: 10.1111/mpp.70100 (PMC12145271; doi:10.1111/mpp.70100)
Supplement: Supplementary file 6 — Table S2. [file MPP-26-e70100-s002.docx]

**Table S2.** Bacterial strains and plasmids used in this study

| **Strain or plasmid** | **Description**^a^ | **Source or reference** |
| --- | --- | --- |
| ***Dickeya oryzae* EC1** | | |
| EC1 | Wild-type soft rot pathogen | Laboratory collection |
| 19 | transposon mutant with insertion at the 36^th^ bp of the coding sequence of *W909_12860*, Gm^r^ | This study |
| 35 | transposon mutant with insertion at the 559^th^ bp of the coding sequence of *W909_00390*, Gm^r^ | This study |
| E174 | transposon mutant with insertion at the 203^th^ bp of the coding sequence of *W909_08520*, Gm^r^ | This study |
| E72 | transposon mutant with insertion at the 2802^th^ bp of the coding sequence of *W909_06710*, Gm^r^ | This study |
| Δ*lrp* | in-frame deletion mutant of *W909_08520* | This study |
| Δ*slyA* | in-frame deletion mutant of *W909_11640* | Laboratory collection |
| Δ*ohrR* | in-frame deletion mutant of *W909_17655* | Laboratory collection |
| Δ*fis* | in-frame deletion mutant of *W909_01320* | Laboratory collection |
| Δ*tzpA* | in-frame deletion mutant of *W909_12860* | Laboratory collection |
| Δ*expI* | in-frame deletion mutant of *W909_00485* | Laboratory collection |
| Δ*speA* | in-frame deletion mutant of *W909_17465* | Laboratory collection |
| Δ*vfmE* | in-frame deletion mutant of *W909_00370* | Laboratory collection |
| Δ*LBD_lrp_* | in-frame deletion mutant of LBD domain of *W909_08520* | This study |
| Δ*HTH_lrp_* | in-frame deletion mutant of HTH domain of *W909*_*08520* | This study |
| Δ*lrp*(*lrp*) | Δ*lrp* carry pBB-*lrp* vector, Ap^r^ | This study |
| Δ*LBD*(*lrp*) | Δ*LBD* carry pBB-*lrp* vector, Ap^r^ | This study |
| Δ*HTH*(*lrp*) | Δ*HTH* carry pBB-*lrp* vector, Ap^r^ | This study |
| Δ*lrp* (*lrp*^D16A^) | site-specific mutagenesis of D16 to alanine for Lrp in *D. oryzae* EC1 | This study |
| Δ*lrp* (*lrp*^L20A^) | site-specific mutagenesis of L20 to alanine for Lrp in *D. oryzae* EC1 | This study |
| Δ*lrp* (*lrp*^L23A^) | site-specific mutagenesis of L23 to alanine for Lrp in *D. oryzae* EC1 | This study |
| Δ*lrp* (*lrp*^I58A^) | site-specific mutagenesis of I58 to alanine for Lrp in *D. oryzae* EC1 | This study |
| Δ*lrp* (*lrp*^G111A^) | site-specific mutagenesis of G111 to alanine for Lrp in *D. oryzae* EC1 | This study |
| Δ*lrp* (*lrp*^T144A^) | site-specific mutagenesis of T144 to alanine for Lrp in *D. oryzae* EC1 | This study |
| Δ*lrp* (*lrp*^T146A^) | site-specific mutagenesis of T146 to alanine for Lrp in *D. oryzae* EC1 | This study |
| ***Escherichia coli*** | | |
| DH5α | *supE44ΔlacU169 (φ80lacZΔM15) hsdR17 recA1 endA1 gyrA96 thi-1 relA1 λpir* | Laboratory collection |
| CC118 | *Δ(ara-leu) araD ΔlacX74 galE galK phoA20 thi-1 rpsE rpoB argE (Am) recA λpir* | Laboratory collection |
| S17-1 | *pro res^-^ mod^+^* integrated copy of RP4; *mob^+^* | Laboratory collection |
| **Plasmids** |  |  |
| pBT20 | Mariner based transposon plasmid, Gm^r^ | Laboratory collection |
| pKNG101 | Suicide vector, Str^r^ | Laboratory collection |
| pBBR1-MCS4 | Low-copy cloning vector, Ap^r^ | Laboratory collection |

^a^Gm^r^, Km^r^, Ap^r^, or Str^r^ = gentamycin, kanamycin, ampicillin, or streptomycin resistant.
