# Supplementary material for: A Lrp/AsnC Family Transcriptional Regulator Lrp Is Essential for the Pathogenicity of Dickeya oryzae
Source: Mol Plant Pathol. 2025 Jun 7;26(6):e70100. doi: 10.1111/mpp.70100 (PMC12145271; doi:10.1111/mpp.70100)
Supplement: Supplementary file 7 — Table S3. [file MPP-26-e70100-s004.docx]

**Table S3.** Primers used in this study

| **Primer name** | **Primer sequence (5’-3’)** | |
| --- | --- | --- |
| **FPNI-PCR** | | |
| FP1 | GTAATACGACTCACTATAGGGCACGCGTGGT NTCGA STWTS GWGTT | |
| FP2 | GTAATACGACTCACTATAGGGCACGCGTGGT NGTCG ASWGA NAWGAA | |
| FP3 | GTAATACGACTCACTATAGGGCACGCGTGGT WGTGN AGWAN CANAGA | |
| FP4 | GTAATACGACTCACTATAGGGCACGCGTGGT AGWGN AGWAN CAWAGG | |
| FP5 | GTAATACGACTCACTATAGGGCACGCGTGGT NGTAW AASGT NTSCA A | |
| FP6 | GTAATACGACTCACTATAGGGCACGCGTGGT NGACG ASWGA NAWGAC | |
| FP7 | GTAATACGACTCACTATAGGGCACGCGTGGT NGACG ASWGA NAWGAA | |
| FP8 | GTAATACGACTCACTATAGGGCACGCGTGGT GTNCG ASWCA NAWGTT | |
| FP9 | GTAATACGACTCACTATAGGGCACGCGTGGT NCAGC TWSCT NTSCTT | |
| FSP1 | GTAATACGACTCACTATAGGGC | |
| FSP2 | ACTATAGGGCACGCGTGGT | |
| SP1  SP2  SP3 | TGTTACGCAGCAGGGCAGTCGC  CCTACTCCCAACATCAGCCGGACTC  TACGGTGACGATCCCGCAGT | |
| **For in-frame deletion vectors** | | |
| lrp-1 | CCTGCAGGTCGACGGATCCTTCCTCATTTCACTCGAACG | |
| lrp-2 | GTTGCTTTGCTTCACTTCTTCGATACGATCAAGATCTTT | |
| lrp-3 | AAAGATCTTGATCGTATCGAAGAAGTGAAGCAAAGCAAC | |
| lrp-4 | CTTATGGTACCCGGGGATCCTAGTAAATGTCGTCTACGTT | |
| lrp-HTH-1 | CCTGCAGGTCGACGGATCCTTCCTCATTTCACTCGAACG | |
| lrp -HTH-2 | AATAAAACCCTGTCGTTCCTTGCGATCGATACGATCAAG | |
| lrp -HTH-3 | CTTGATCGTATCGATCGCAAGGAACGACAGGGTTTTATT | |
| lrp -HTH-4 | CTTATGGTACCCGGGGATCCTCTTCTGTGTATTCCTGGCT | |
| lrp -LBD-1 | CCTGCAGGTCGACGGATCCTACTTTGACAATCGGCTGCG | |
| lrp -LBD-2 | TCAGCGGGTCTTGATGACCACGGTTGAGGGTGATTTCAAC | |
| lrp -LBD-3 | GTTGAAATCACCCTCAACCGTGGTCATCAAGACCCGCTGA | |
| lrp -LBD-4 | CTTATGGTACCCGGGGATCCTAGTAAATGTCGTCTACGTT | |
| **For complemention^a^** | | |
| D16-2 | GTTGCGTGCGATACGATCAA | |
| D16-3 | TTGATCGTATCGCACGCAAC | |
| L20-2 | TGcaaTTCGTTTGCGATGTTG | |
| L20-3 | CAACATCGCAAACGAAttgCA | |
| L23-2 | CCCGTCTTTTTGTGCTTCGTT | |
| L23-3 | AACGAAGCACAAAAAGACGGG | |
| I58-2 | ATAGCCATTTGCAAAACCCTG | |
| I58-3 | CAGGGTTTTGCAAATGGCTAT | |
| G111-2 | GTCAAAATCTGCAGAAACCAG | |
| G111-3 | CTGGTTTCTGCAGATTTTGAC | |
| T144-2 | CGTGAACGATTGCCGCACCT | |
| T144-3 | AGGTGCGGCAATCGTTCACG | |
| T146-2 | GATACCCGCTGCTATGTGGT | |
| T146-3 | ACCACATAGCAGCGGGTATC | |
| lrp-F | tcgacggtatcgataagcttATGGTAGACACGAAAAAGCG | |
| lrp-R | gctctagaactagtggatccTCAGCGGGTCTTGATGACCA | |
| **For amplification of promoter fragments in EMSA analysis** | | |
| ohrR-F | GTGAGTCGTATTCGGCCAGA | |
| ohrR-R | GGCTGCCACTCTATATCGCT | |
| pehK-F | AGTAAACATCTTGCGCGATC | |
| pehK-R | TCATGGTGGTGATCCTGTAT | |
| pehX-F | CAGGAAAGCCTGTGGCAAAG | |
| pehX-R | TACCTCACAGCCCATCGCCT | |
| prtX-F | AGTTCGGCTGATTTTCTGGT | |
| prtX-R | CGCAATAATCAGCTTTTTCA | |
| prtG-F | ATCAGACTATGCTGCCTGAA | |
| prtG-R | CACGCTTCCTCCATGAAATT | |
| fis-F | GTTGTTCGAACATAGTTCTG | |
| fis-R | AATCCAGCATTATCTGGACA | |
| zmsA-F | GCTGCTGTGGTTTCAGCTAT | |
| zmsA-R | ATATTCCCTCTCACAAAAGG | |
| zmsK-F | GAACTGGAAACGCAACAACT | |
| zmsK-R | GTTGCTCATGCGCTCACCT | |
| tzpA-F | GGTGATACCGTCTGTTTCTC | |
| tzpA-R | CGCTAATCAAAGAACAATTCT | |
| 08510-F | CATATTTATCGCCAGGCCAT | |
| 08510-F | CAGGTGCAGGTCGGTTCCAG | |
| **For RT-qPCR** | | |
| qPCR-16S-F | | CCAGGTGTAGCGGTGAAATGC |
| qPCR-16S-R | | CGGAAGCCACGGTTCAAGAC |
| qPCR-zmsA-F | | CGTCAGTAGAGTCGGAGA |
| qPCR-zmsA-R | | CTGCTGTCGTGGCTATAC |
| qPCR-zmsB-F | | CTGACACCACGAACACTA |
| qPCR-zmsB-R | | CAACGAACGACCATCATC |
| qPCR-zmsC-F | | GGCTACAGCGATAAGGTTA |
| qPCR-zmsC-R | | GCAGGTTGGAATACATACG |
| qPCR-zmsD-F | | ACTCTGTTAGCACCACTG |
| qPCR-zmsD-R | | TGAGCAGACAGGCAATAG |
| qPCR-zmsE-F | | CTTATCGCTCGTCATTCAC |
| qPCR-zmsE-R | | GTAATGCCGCTACCTTCA |
| qPCR-zmsG-F | | GAGGATGAACTGGCAGAA |
| qPCR-zmsG-R | | GGATGGCAATTCAGATAACG |
| qPCR-zmsI-F | | GGGAAAGGAAGTGAAATGC |
| qPCR-zmsI-R | | ATTATCCAGTGACCAGCG |
| qPCR-zmsJ-F | | GTTGGTGATGTCGTTATGGA |
| qPCR-zmsJ-R | | GACCGCCGAACAGAATAC |
| qPCR-zmsK-F | | GTCTCTGCTACAAGGTAATC |
| qPCR-zmsK-R | | CTCCAACGCTGACAATAC |
| qPCR-zmsN-F | | CAGCGTATCAACCTCAATG |
| qPCR-zmsN-R | | CCAACCAGAATATCGTTACC |
| qPCR-zmsP-F | | CTTGGATTGCTGTTGCTAAG |
| qPCR-zmsP-R | | TTAACTTCGTCGCCTTCC |
| qPCR-prtA-F | | CCTCCAGCAGCAGTTATG |
| qPCR-prtA-R | | CCTCCTCAAGCGAGTAAG |
| qPCR-prtB-F | | CGGGCGATATACCTTCAC |
| qPCR-prtB-R | | GAACTGGCGGGAATCTTC |
| qPCR-prtD-F | | CGGAAGGTCATCTCAGTG |
| qPCR-prtD-R | | CTTACCAGAACCAGAGGC |
| qPCR-prtF-F | | TGATACCCAGACGGTAGG |
| qPCR-prtF-R | | CCGGGTCTGATTATCCAG |
| qPCR-prtG-F | | TCGTCCGGTAGCTATACC |
| qPCR-prtG-R | | CTCCAGCGTATAGGATGG |
| qPCR-prtX-F | | GAGCAGTCTGAGATTACC |
| qPCR-prtX-R | | CCAGGTTGTGTTATCCAG |
| qPCR-pehK-F | | GTACTGGAGGACTGTGAG |
| qPCR-pehK-R | | GTCGTTATCCACCGTCAG |
| qPCR-pehX-F | | CTATGCCGACGGTACAAC |
| qPCR-pehX-R | | CTGGTATTGAGCGTGGTG |
| qPCR-fliA-F | | CGTTACTGGAAGGGAATGAG |
| qPCR-fliA-R | | CCTGGTAGTAGAGAGTCAAC |
| qPCR-fliD-F | | CGGTTGGGTATCAATGAGGA |
| qPCR-fliD-R | | TGAAGCGGAAGTGGCATATC |
| qPCR-fliG-F | | CCGAGTTGCTGGAAGTATTG |
| qPCR-fliG-R | | CTTCACCCAGTGCTTTAACC |
| qPCR-fliM-F | | GCCCTATGATCCCAATACAC |
| qPCR-fliM-R | | GCACCAACGGTAATATCAGG |
| qPCR-flhC-F | | GGGAACAGAACATCCACTCT |
| qPCR-flhC-R | | GCGATAAGCCTTGATAACCG |
| qPCR-fis-F | | CTCTCAGGCTCAGGTAAC |
| qPCR-fis-R | | GCCAGTACCAGCTCATAC |
| qPCR-ohrR-F | | CTGAGTGATGACGCCTTC |
| qPCR-ohrR-R | | CCAGATACTGTGGGTACG |
| qPCR-slyA-F | | GATATGGCTGATCCGATC |
| qPCR-slyA-R | | GGCTAACTCATCCACTTC |
| qPCR-tzpA-F | | CAGCAGATGGCATTAAGC |
| qPCR-tzpA-R | | CGGGCTAAGATTGAGTTG |
| qPCR-lrp-F | | TGTTCGTTGAAATCACCCTC |
| qPCR-lrp-R | | AGAAACCAGATGACACTCCT |
| qPCR-vfmE-F | | CGTCTCTTCAACCAGGTG |
| qPCR-vfmE-R | | GGTGAGCGACCAATTAGG |
| qPCR-expI-F | | CCGAAGGGAATTATATTGAGTC |
| qPCR-expI-R | | AATCATAGCGAGGAATAACG |
| qPCR-speA-F | | GTATGACCCGTACCGTTATC |
| qPCR-speA-F | | AATGACCAGATAGACCTTGTG |
| **For protein expression vectors** | | |
| pGEX-*lrp*-F | | tgttccaggggcccctgggatccATGGTAGACACGAAAAAGCG |
| pGEX-*lrp*-R | | ctcgagtcgacccgggaattcTCAGCGGGTCTTGATGACCA |

^a^ The red font indicates the position of mutation.
